# The Transcription Factor WFZP Interacts with the Chromatin Remodeler TaSYD to Regulate Root Architecture and Nitrogen Uptake Efficiency in Wheat

**DOI:** 10.1002/advs.202416433

**Published:** 2025-02-24

**Authors:** Dejie Du, Zhaoju Li, Zihao Jiang, Jun Yuan, Xiangyu Zhang, Huanhuan Zhao, Lulu Tian, Yunjie Liu, Renhan Li, Fei He, Xiongtao Li, Wensheng Ke, Lingling Chai, Jie Liu, Mingming Xin, Yingyin Yao, Qixin Sun, Jiewen Xing, Zhongfu Ni

**Affiliations:** ^1^ Frontiers Science Center for Molecular Design Breeding Key Laboratory of Crop Heterosis and Utilization (MOE) Key Laboratory of Crop Genetic Improvement China Agricultural University Beijing 100193 China

**Keywords:** lateral root number, nitrogen uptake efficiency, TaSYD, WFZP, wheat

## Abstract

The root architecture is crucial for the robust growth and nutrient absorption in cereals. However, it is urgent to identify the factors that simultaneously optimize root architecture and nutrient utilization in wheat. In this study, a beneficial role of the class II AP2/ERF transcription factor WHEAT FRIZZY PANICLE (WFZP) on lateral root number (LRN), root length (RL), and nitrogen utilization is revealed. In addition, interactors of WFZP including TaSYD are identified, as a subunit of the chromatin remodeling complex. The *Tasyd* mutants show a significant reduction in LRN, RL, and nitrogen uptake efficiency, resembling the phenotype of *wfzp* mutants. Furthermore, it is revealed that the WFZP‐TaSYD module promotes the expression of root development and nitrate uptake‐related genes by modulating chromatin accessibility and histone modifications. Finally, an elite allele (*WFZP‐A‐I*) associated with improved LRN and thousand‐grain weight (TGW) is identified. Hence, these findings not only unveil the mechanisms underlying the coordination of root development and nitrogen uptake efficiency, but also provide valuable targets for breeding high‐yield crops.

## Graphical abstract

Root development and nitrogen uptake efficiency are vital for crop productivity. Du, Li, Jiang, et al. show that the wheat transcription factor WFZP and the chromatin remodeler TaSYD form a complex within the nucleus, thereby activating the expression of genes associated with root development and nitrate transporters, ultimately enhancing both root growth and nitrogen uptake efficiency.

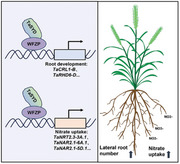

## 1 Introduction

The root system is essential for water and nutrient absorption from the surrounding environment, enabling plants to maintain their physiological processes and achieve optimal growth.^[^ ^1^ ^]^ The monocot cereals, such as wheat and rice, possess a complex root system comprising various root types, including primary roots, lateral roots (LRs), and adventitious roots.^[^ ^2^ ^]^ Among these, LRs are a key determinant of root systems architecture and significantly affect the efficiency of resource acquisition.^[^ ^3^, ^4^ ^]^ However, the molecular mechanisms underlying the regulation of these traits in wheat remained largely elusive.

To date, molecular genetic studies have identified several crucial genes that regulate lateral root initiation in Arabidopsis and crown root formation in rice. For instance, Aux/IAA28‐dependent auxin signaling module positively activates the expression of *GATA23*, which is responsible for founder cell specification in the lateral root.^[^ ^5^ ^]^ Two auxin response factors, AUXIN RESPONSE FACTOR (ARF)7 and ARF19, directly activate the transcription of *LATERAL ORGAN BOUNDARIES DOMAIN* (*LBD*)*16* and *LBD29* thereby facilitating lateral root formation in Arabidopsis.^[^ ^6^, ^7^, ^8^, ^9^ ^]^ In rice, *ROOTLESS1* (*CRL1*), which encodes a protein containing an LBD domain, plays a key role in the initiation of crown roots and lateral roots.^[^ ^6^, ^7^ ^]^ Additionally, rice WUSCHEL‐related Homeobox 11 (WOX11) is responsive to auxin and cytokinin, and it interacts with CRL1 to synergistically enhance the expression of *Cytokinin oxidase/dehydrogenases 4*, which is essential for the development and growth of crown roots.^[^ ^7^ ^]^

Recently, several genes regulating the root architecture of wheat have been identified as well. *VERNALIZATION1* (*VRN1*), a critical regulator of flowering, also influences root architecture in wheat and barley.^[^ ^8^ ^]^ Near Isogenic Lines (NILs) carrying the spring allele displayed a significantly greater number of roots (5.3%) at a depth of 60–80 cm compared to the winter types.^[^ ^8^ ^]^ The LBD gene *TaMOR* (*MORE ROOT in wheat*) regulates crown root initiation by interacting with the auxin response factor TaARF5 to induce the expression of the auxin transporter gene *PIN2*. The *mor* mutants exhibited reduced or absent crown roots, dwarfism, fewer grains, and lodging due to inhibited root initiation.^[^ ^9^ ^]^ Dosage variations in monocot‐specific *12‐OXOPHYTODIENOATE REDUCTASE* genes (*OPRIII*) significantly influence wheat root architecture and grain yield under water‐limited conditions, with loss‐of‐function mutants possessing longer seminal roots, while increased *OPRIII* dosage results in reduced seminal root growth and earlier lateral root development.^[^ ^10^ ^]^ The *NPF2.12* gene in wheat, a homolog of Arabidopsis nitrate transceptor *NRT1.6*, shows promoter variations linked to decreased expression under low nitrate availability. The *npf2.12* mutants exhibit increased nitrogen utilization and improved root growth, and the elite haplotypes of *NPF2.12* have been selected in wheat and barley during domestication.^[^ ^11^ ^]^

In eukaryotes, the regulation of gene transcription is finely tuned through dynamic alterations in chromatin accessibility in response to developmental and environmental signals.^[^ ^12^, ^13^ ^]^ Switch defective/sucrose non‐fermentable (SWI/SNF) chromatin remodeling complexes are essential for regulating nucleosome occupation and positioning.^[^ ^14^, ^15^ ^]^ In recent years, multiple homologs of several SWI/SNF subunits have been identified in Arabidopsis: two canonical ATPase subunits, SPLAYED (SYD) and BRAHMA (BRM);^[^ ^16^ ^]^ four SWI3 subunits (SWI3A–SWI3D);^[^ ^17^, ^18^, ^19^ ^]^ two actin‐related proteins (ARP4 and ARP7);^[^ ^20^ ^]^ two BRM‐associated factors (SWP73A and SWP73B);^[^ ^21^ ^]^ and BUSHY (BSH, an ortholog of SNF5/INI1).^[^ ^22^ ^]^ Mutants of *SYD* in Arabidopsis exhibit pleiotropic developmental defects, including slow growth, small and upward‐curled leaves and bracts, aberrant flower development, and sterility.^[^ ^23^ ^]^ Furthermore, SYD interacts with LFR to activate *AG* transcription, a gene crucial for determining floral organ identity through regulating nucleosome occupancy, chromatin loop formation, histone modification, and Pol II enrichment on the *AG* locus.^[^ ^24^ ^]^ However, the mechanism details of SWI/SNF chromatin remodeling ATPases in regulating root development and nutrient absorption remain elusive.

Here, we reported the novel role of WFZP in regulating root development and nitrogen uptake efficiency in wheat. WFZP directly interacts with SWI/SNF chromatin remodeling ATPases TaSYD, thereby co‐regulating root development and nitrate uptake‐related genes by modulating chromatin accessibility and histone modifications. Furthermore, haplotype analysis indicates that a G‐to‐A substitution in the *WFZP‐A* promoter (referred to as *WFZP‐A‐I*) is significantly associated with improved LRN and TGW, which highlights the critical role of the WFZP‐SYD module in nutrient acquisition and yield determination.

## 2 Results

### 2.1 WFZP Controls RL and LRN in Wheat

**Figure 1:**
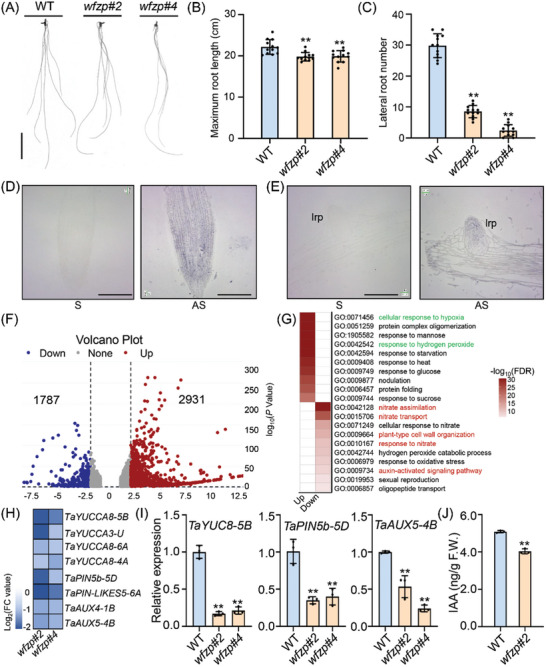
WFZP is a positive regulator for root development. A) Root phenotypes of WT and *wfzp* mutants for 10 days’ hydroponics. Scale bars, 5 cm. B,C) Maximum RL (B) and LRN (C) of WT and *wfzp* mutants in seeding stage. Values are means ± s.d. (Student's *t*‐test, ^**^ *p* < 0.01, *n* = 12). D,E) In situ hybridization assays showing the transcripts of *WFZP* in root tips (D) and lateral root primordia (E). The sense probe was used a negative control. lrp, lateral root primordia; S, the sense probe; AS, the antisense probe. Scale bars, 200 µm. F) Volcano plot showing the numbers of DEGs shared among WT and *wfzp#2* mutant. 1787 and 2931 represent the numbers of down‐regulated and up‐regulated genes in *wfzp#2* compared to WT, respectively. G) GO analysis of co‐DEGs shared by *wfzp#2* and *wfzp#4* compared to WT. H) Heatmap revealing the expression levels of the auxin synthesis and transport‐related genes in *wfzp* mutants compared to those in WT. I) Validation of the selected auxin synthesis and transport‐related genes using qRT‐PCR. The transcript levels of WT were set as 1. Values are means ± s.d. (Student's *t*‐test, ^**^ *p* < 0.01, n = 3). J) Comparison of IAA content in the roots of *wfzp#2* mutant and WT after 10 days’ hydroponic growth. Values are means ± s.d. (Student's *t*‐test, ^**^ *p* < 0.01, *n* = 3).

In previous studies, we generated *wfzp‐a/d* mutants through CRISPR/Cas9‐mediated gene editing in Fielder background.^[^ ^25^ ^]^ Given its high expression in roots, we speculated that WFZP might play a role in shaping root architecture.^[^ ^25^ ^]^ To test this hypothesis, two *wfzp‐a/d* mutants, hereafter named *wfzp#2* and *wfzp#4*, were selected for phenotypic analyses. Both *wfzp* mutants exhibited a 10.2–10.6% reduction in primary RL compared to wild type (WT) after 10 days of hydroponics (**Figure** **1**A,B). Moreover, the two *wfzp* lines showed a significant decrease in LRN, ranging from 2.4 to 8.5, compared to WT with an average of 29.8 (Figure 1A,C). We also measured the RL of the seedlings in pipes at 20 days after germination and found that the root length of *wfzp#4* was significantly shorter than that of WT (Figure S1, Supporting Information). Additionally, in situ hybridization revealed strong signals of *WFZP* at root apical meristem and lateral root primordium (Figure 1D,E), suggesting a potential function of WFZP in primordial cell division. These results provide evidence that WFZP is crucial for promoting both RL and LRN.

To identify the downstream genes of WFZP, a transcriptome analysis was conducted using the roots of *wfzp* mutants and WT plants. Compared to WT, a total of 4718 differentially expressed genes (DEGs) were identified in *wfzp* mutants, with 2931 up‐regulated and 1787 down‐regulated (Figure 1F). Gene ontology (GO) analysis revealed that these DEGs, especially the down‐regulated genes, were enriched in multiple biological processes, including “nitrate assimilation and transport”, “plant‐type cell wall organization”, “response to nitrate” and “auxin‐activated signaling pathway” etc. (Figure 1G). Auxin is well known to regulate LR initiation.^[^ ^26^, ^27^ ^]^ Previous studies have shown that LR initiation involves auxin accumulation controlled by auxin transport systems and signaling pathways mediated by ARF and Aux/IAA proteins.^[^ ^28^, ^29^ ^]^ In this study, we found that a set of auxin synthesis and transport‐related genes were significantly decreased in *wfzp* mutants (Figure 1H). Consistently, qRT‐PCR assays confirmed that the transcript levels of *TaYUC8‐5B*, *TaPIN5b‐5D*, and *TaAUX5‐4B* were all significantly down‐regulated in *wfzp* mutants (Figure 1I). In addition, we measured the endogenous auxin levels in the roots of both the WT and *wfzp#2* mutant after 10 days of hydroponics. The results revealed that the IAA levels are significantly lower in the *wfzp#2* mutant compared to the WT (Figure 1J). Taken together, these findings indicate that WFZP plays a crucial role in the regulation of root development, possibly by modulating auxin synthesis and transport.

### 2.2 WFZP Physically Interacts with SWI/SNF Chromatin Remodeler TaSYD

To further investigate the regulatory mechanisms of WFZP, we performed a yeast two‐hybrid (Y2H) assay to identify its interacting proteins. Considering that WFZP‐A has self‐activation activity (Figure S2, Supporting Information), we used a truncated version of WFZP‐A, referred to as WFZP‐A‐P3, as a bait, and finally obtained 32 candidate interactors (Table S6, Supporting Information). Among them, TraesCS7B02G110600 was annotated as a chromatin remodeling complex subunit R3 (CHR3), suggesting its potential role in regulating chromatin accessibility. Sequence analysis revealed that TraesCS7B02G110600 shares high amino acid identities with Arabidopsis SYD (AT2G28290), thus hereafter named *TaSYD‐B*. The three homoeologs of *TaSYD‐A* (*TraesCS7A02G203600*), *TaSYD‐B* (*TraesCS7B02G110600*) and *TaSYD‐D* (*TraesCS7D02G206700*) of the *TaSYD* gene in Chinese Spring (CS) were 97.04% identical in protein sequences (Figure S3, Supporting Information), indicating the crucial and conserved role of TaSYD.

**Figure 2:**
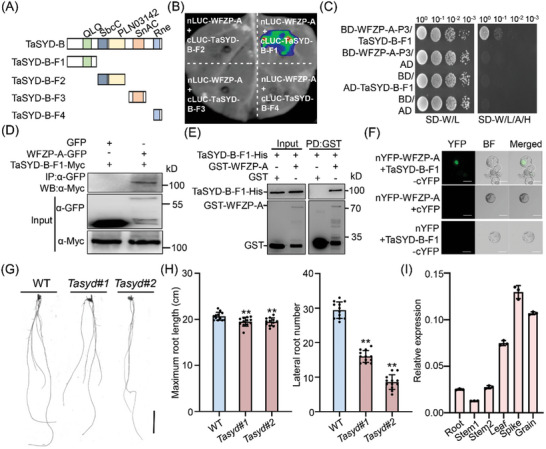
TaSYD‐B directly interacts with WFZP‐A. A) Schematic diagram showing TaSYD‐B and different truncated protein fragments. QLQ, SbcC, PLN03142, SnAC and Rne represent distinct domains. B) Firefly luciferase (LUC) complementation imaging (LCI) assay showing that TaSYD‐B‐F1 interacts with WFZP‐A in *N. benthamiana* leaves. C) Yeast two‐hybrid (Y2H) showing the interaction between WFZP‐A‐P3 and TaSYD‐B‐F1. The transformed yeast cells were grown on synthetic dextrose (SD) medium lacking Trp and Leu (SD‐L/W), and selected on SD lacking Trp, Leu, Ade and His (SD‐L/W/H/A). AD, pGADT7 vector; BD, PGBKT7 vector. D) Co‐IP assay showing that the TaSYD‐B‐F1 associates with WFZP‐A in *N. benthamiana* leaves. Proteins were immunoprecipitated (IP) with GFP beads. The immunoblot assays were performed using anti‐GFP and anti‐Myc antibodies. WB, Western Blot. E) Pull‐down assay showing that WFZP‐A interacts with TaSYD‐B‐F1 in vitro. GST was used as a negative control. Blots were detected with anti‐His and anti‐GST antibodies, respectively. PD, pull‐down. F) Bimolecular fluorescence complementation (BiFC) showing the interaction between WFZP‐A and TaSYD‐B‐F1 in the nucleus of protoplasts derived from wheat roots. nYFP, N‐terminal part of yellow fluorescent protein (YFP); cYFP, C‐terminal part of YFP. G) Root phenotypes of WT and *Tasyd* mutants after 10 days’ hydroponic growth. Scale bars, 5 cm. #1 and #2 represent the line name of *Tasyd* mutants. H) Statistical analyses of maximum RL (left) and LRN (right) between WT and *Tasyd* mutants. Values are means ± s.d. (Student's *t*‐test, ^**^ *p* < 0.01, *n* = 12). I) The expression pattern of *TaSYD* in different tissues. Stem1, 1–1.5 cm; Stem2, 2–2.5 cm. Values are means ± s.d. (*n* = 3).

*TaSYD‐B* encodes a nuclear‐localized protein of 3382 amino acids with five domains, namely QLQ, SbcC, PLN03142, SnAC, and Rne (**Figure** **2**A; Figure S3, Supporting Information). To identify the domain responsible for the interaction, the luciferase complementation imaging assays (LCI) were performed using truncated proteins of TaSYD‐B. Results revealed that the TaSYD‐B‐F1, containing the QLQ domain, was required for the physical interaction between TaSYD and WFZP‐A (Figure 2B). The Y2H assay confirmed that TaSYD‐B‐F1 strongly interacted with WFZP‐A‐P3 (Figure 2C). Furthermore, the coimmunoprecipitation (Co‐IP) assay, by transiently co‐expressing *WFZP‐A‐GFP* and *TaSYD‐B‐F1‐Myc* in plants showed that WFZP‐A‐GFP, but not the GFP alone, can interact with TaSYD‐B‐F1‐Myc in planta (Figure 2D). Pull‐down assays demonstrated that GST‐WFZP‐A, but not GST itself, specifically pulled down TaSYD‐B‐F1‐His protein in vitro (Figure 2E; Figure S4, Supporting Information). Additionally, bimolecular fluorescence complementation (BiFC) also revealed that WFZP‐A interacted with TaSYD‐B‐F1 in the nucleus of protoplasts derived from wheat roots (Figure 2F). Taken together, these results suggest that WFZP‐A interacts with TaSYD‐B both in vitro and in vivo, with the QLQ domain important for their interaction.

As a WFZP‐interacting partner, TaSYD may also play a role in the regulation of LRN and RL. To test this hypothesis, we employed the CRISPR/Cas9 editing strategy to generate two independent *Tasyd‐a/b* mutants (#1 and #2) in the Fielder background by simultaneously knocking out A‐ and B‐homeologs while retaining intact *TaSYD‐D* (Figure S5, Supporting Information). Phenotypic analyses revealed that both *Tasyd‐a/b* mutants exhibited significantly reduced LRN and RL than WT, similar to the *wfzp* mutant after 10 days of hydroponics (Figure 2G,H). The qRT‐PCR results exhibited that *TaSYD* was constitutively expressed in various tissues of wheat including young roots (Figure 2I). In situ hybridization assays further showed that *TaSYD* was primarily expressed in root tips and lateral root primordia (Figure S6, Supporting Information), suggesting a potential role for TaSYD in the regulation of root development.

### 2.3 Transcriptome Profiling of WFZP and TaSYD Co‐Regulated Genes in Roots

**Figure 3:**
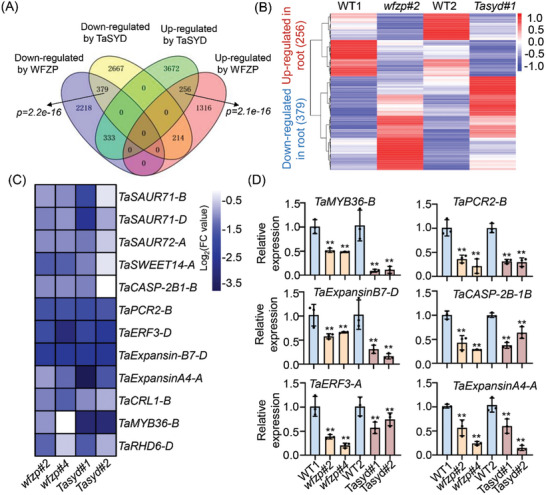
Genome‐wide profiling of DEGs regulated by WFZP and TaSYD. A) Venn diagram showing the overlap of WFZP‐regulated and TaSYD‐regulated genes. Genes in the overlapping region were identified as co‐regulated genes of WFZP and TaSYD during root development. The hypergeometric test was performed to calculate the *p* value. B) Heatmap revealing the fold changes of co‐regulated genes in *wfzp#2* and *Tasyd#1* mutants. WT1 was the contrast of *wfzp#2*, WT2 was the contrast of *Tasyd#1*. The scale bar represents log_2_FC of the average expression level of each gene in three biological replicates. C) Heatmap representing the well‐characterized root development‐related genes co‐regulated by WFZP and TaSYD. D) qRT‐PCR assay confirming the expression of some selected genes listed in (C) in different samples. The transcript levels of WT1 and WT2 were separately set as 1. Values are means ± s.d. (Student's *t*‐test, ^**^ *p* < 0.01, *n* = 3).

To identify genes regulated by the WFZP‐TaSYD complex during root development, we examined the transcriptome of *wfzp*, *Tasyd*, and WT roots at 10 days of hydroponics. Compared with WT, a total of 4718 and 7521 genes were identified as DEGs (FC > 2, Padj < 0.05) for *wfzp* and *Tasyd*, respectively. The identified DEGs in each sample were referred to as WFZP‐regulated genes and TaSYD‐regulated genes (**Figure** **3**A,B). Several randomly selected DEGs shared by WFZP and TaSYD were further validated by qRT‐PCR, and the results were consistent with the transcriptome data (Figure S7, Supporting Information). Among them, 379 genes were co‐downregulated and 256 genes were co‐upregulated by the WFZP‐TaSYD module (Figure 3A,B).

Our transcriptome analysis revealed that WFZP and TaSYD co‐regulate several well‐characterized genes associated with root development. For example, auxin‐responsive genes including *TaSAUR71‐B*, *TaSAUR71‐D* and *TaSAUR72‐A*, sugar transporter *TaSWEET14‐A*, aspartic protease *TaCASP‐2B1‐B*, cell number regulator *TaPCR2‐B*, expansin gene *TaExpansin‐B7‐D* and *TaExpansinA4‐A*, and several key transcriptional factors including *TaERF3‐D*, *TaCRL1‐B*, *TaMYB36‐B* and *TaRHD6‐D* (Figure 3C). Furthermore, qRT‐PCR assays confirmed that some of these genes were significantly downregulated in both *wfzp* and *Tasyd* mutants compared to WT (Figure 3D), suggesting that the WFZP‐TaSYD complex plays an essential role in activating the genes involved in root development.

### 2.4 *TaCRL1* and *TaRHD6* Are the Downstream Targets of WFZP‐TaSYD Complex

**Figure 4:**
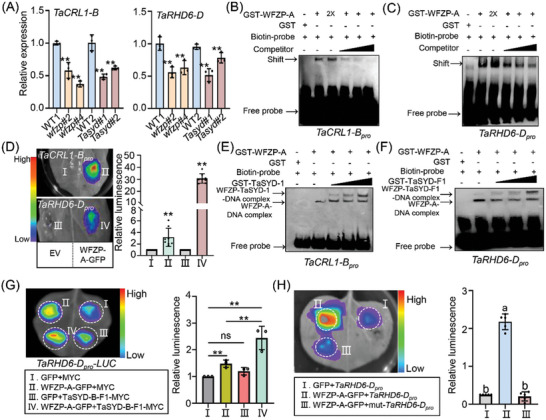
WFZP‐TaSYD complex directly activates the expression of *TaCRL‐B* and *TaRHD6‐D* through promoter binding. A) qRT‐PCR assay showing the expression of *TaCRL1‐B* (left) and *TaRHD6‐D* (right) in *wfzp* and *Tasyd* mutants compared to WT. Values are means ± s.d. (Student's *t*‐test, ^**^ *p* < 0.01, *n* = 3). B,C) EMSA assays showing the direct binding of GST‐WFZP‐A to *TaCRL1‐B* (B) and *TaRHD6‐D* (C) promoters. The black triangles indicate an increase in the number of competitive probes, the above arrow head indicates specific bands. D) Transient transcriptional assays showing that WFZP‐A notably activates the expression of *TaCRL1‐B* and *TaRHD6‐D* genes in *N. benthamiana* leaves. Left panels show representative leaf images, and the right column represents the statistical analyses of relative luminescence intensities. Combination I and combination III are the controls for combination II and combination IV, respectively. Values are means ± s.d. (Student's *t*‐test, ^**^ *p* < 0.01, *n* = 8). E,F) TaSYD‐B‐F_1_ retarded the binding shifts of the *WFZP‐A‐TaCRL1‐B‐pro* (E) and *WFZP‐A‐TaRHD6‐D‐pro* (F). The angles indicate an increase in the number of GST‐TaSYD‐1. G) Transient expression assays illustrating that the combination of WFZP‐A and TaSYD‐B‐F1 could effectively activate the expression of LUC reporter gene driven by the *TaRHD6‐D* native promoter. Mean values in combination I was set to 1. Values are means ± s.d. (Student's *t*‐test, ^**^ *p* < 0.01, *n* = 3). H) The mutated promoter almost completely weakened the activation effect of WFZP‐A. Values are means ± s.d. (^**^ *p* < 0.01, *n* = 5, one way ANOVA, Tukey's HSD test).

In previous studies, the Crown rootless 1 (CRL1) and ROOT HAIR DEFECTIVE 6 (RHD6) were demonstrated to play critical roles in initiating adventitious root primordia in rice and root hairs in Arabidopsis.^[^ ^6^, ^30^ ^]^ We found a significant reduction in the expression of *TaCRL1‐B* and *TaRHD6‐D* in both *wfzp* and *Tasyd* mutants compared to the WT, as supported by our transcriptome data and qRT‐PCR assay (Figure 3C; **Figure** **4**A). It is reported that WFZP can bind to GCC‐box cis‐elements.^[^ ^25^ ^]^ Through scanning the promoters, we found multiple highly confident GCC‐box cis‐elements in the 2kb‐promoters of *TaCRL1‐B* and *TaRHD6‐D* (Figures S9–S11, Supporting Information), suggesting that WFZP may directly regulate their expression. Electrophoretic mobility shift assay (EMSA) demonstrated that WFZP directly binds to all tested GCC‐containing motifs in the promoters of *TaCRL1‐B* and *TaRHD6‐D* (Figure 4B,C). In addition, transient expression assays revealed that WFZP could efficiently activate *TaCRL1‐B* and *TaRHD6‐D* expressions (Figure 4D). Interestingly, the EMSA assay also indicated that WFZP and TaSYD could form complexes in association with the promoters of *TaCRL1‐B* or *TaRHD6‐D* (Figure 4E,F). Furthermore, the activation of *TaRHD6‐D* was more pronounced by WFZP‐A upon co‐expression of TaSYD‐B in *N. benthamiana* leaves (Figure 4G). We also created motif‐mutated versions of the *TaRHD6‐D* promoter, which almost completely diminished the activating effect of WFZP‐A on *TaRHD6‐D* (Figure 4H). These results showed that WFZP and TaSYD can synergistically activate *TaCRL1‐B* and *TaRHD6‐D* expression and thereby regulate root development.

### 2.5 WFZP‐TaSYD Module Regulates Nitrate Utilization in Wheat

**Figure 5:**
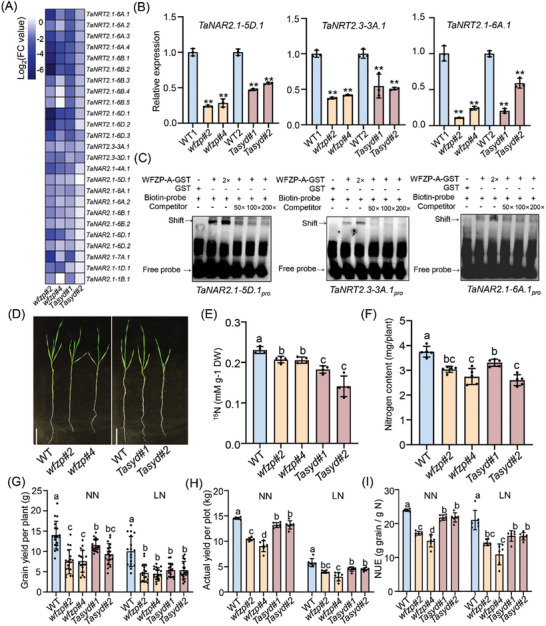
WFZP‐TaSYD complex positively regulates N uptake and NUE. A) Hierarchical clustering analysis of high‐affinity nitrate transporter‐related genes co‐regulated by WFZP and TaSYD. B) qRT‐PCR assays confirming the expression of selected co‐regulated genes, such as *TaNAR2.1‐5D.1*, *TaNRT2.3‐3A.1* and *TaNRT2.1‐6A.1*. WT1 was the contrast of *wfzp*, WT2 was the contrast of *Tasyd*. C) EMSAs confirming that WFZP could bind directly to the GCC‐containing promoter sequences of *TaNAR2.1‐5D.1* (left), *TaNRT2.3‐3A.1* (middle) and *TaNRT2.1‐6A.1* (right). D) The phenotypes of *wfzp* and *Tasyd* mutants were compared with WT plants treated with ^15^N‐nitrate for 2 weeks. Scale bars, 5 cm. E) Accumulation of ^15^N in 2‐week‐old WT, *wfzp* and *Tasyd* seedlings grown with the 2.5 mm ^15^N. Values are means ± s.d. (^**^ *p* < 0.01, *n* = 4, one way ANOVA, Tukey's HSD test). F) Total N content of WT, *wfzp* and *Tasyd* mutants grown hydroponically with 2.5 mm KNO_3_ for 2 months. Values are means ± s.d. (^**^ *p* < 0.01, *n* = 5, one way ANOVA, Tukey's HSD test). G–I) Grain yield per plant (G), actual yield per plot (H), NUE (I) of WT, *wfzp* mutants (*wfzp#2*/ *wfzp#4*) and *Tasyd mutants* (*Tasyd#1*/*Tasyd#2*) under LN, NN conditions in the field trial. Values are the means ± s.d. (^**^ *p* < 0.01, *n* = 18 for E, *n* = 6 for G and H, one way ANOVA, Tukey's HSD test).

Previous studies have shown that *OsNRT* and *OsNAR* in rice are essential for nitrogen utilization and crop yield.^[^ ^31^, ^32^, ^33^, ^34^ ^]^ In transcriptome data, we observed that a specific set of nitrate transport and reduction‐related genes were simultaneously down‐regulated in *wfzp* and *Tasyd* mutant (**Figure** **5**A), implying that the WFZP‐TaSYD module may play a critical role in the regulation of nitrogen utilization. Next, three genes, *TaNAR2.1‐5D.1*, *TaNRT2.3‐3A.1* and *TaNRT2.1‐6A.1* were selected for qRT‐PCR assay, and the patterns were similar to those in the transcriptome (Figure 5B). In addition, several cis‐elements predicted to be recognized by WFZP were located on the promoters of *TaNAR2.1‐5D.1*, *TaNRT2.3‐3A.1* and *TaNRT2.1‐6A.1* (Figures S12–S15, Supporting Information). The binding specificity of WFZP to these motifs was then verified by EMSA (Figure 5C). Transient expression assays revealed that the expressions of these genes were efficiently activated by WFZP‐A (Figure S16, Supporting Information). These results demonstrated that WFZP‐TaSYD might regulate nitrogen utilization by directly transactivating the genes related to nitrate uptake and assimilation.

To test this hypothesis, we investigated the role of WFZP in regulating nitrogen absorption. With a ^15^NO_3_^−^ measurement, we found that the ^15^N accumulations in *wfzp* and *Tasyd* mutants were both significantly lower than those in WT (Figure 5D,E). Moreover, the total N contents in both *wfzp* and *Tasyd* mutants were decreased compared with those of WT (Figure 5F). These results indicated that the WFZP‐TaSYD module is required for nitrogen uptake. To investigate the role of WFZP in nitrogen use efficiency (NUE), we conducted field trials for *wfzp* and *Tasyd* mutants under low nitrogen (LN) and normal nitrogen (NN) conditions. The representative plants of each genotype are shown in Figure S17, Supporting Information. Grain yield per plant and per plot of both *wfzp* and *Tasyd* mutants exhibited a significant decrease under both conditions compared with WT (Figure 5G,H). Nitrogen uptake efficiency (NUpE) and nitrogen utilization efficiency (NUtE) are' two major factors determining the overall NUE.^[^ ^35^ ^]^ Therefore, we quantified NUE, NupE, and NUtE in *wfzp* and *Tasyd* mutants at the mature stage under LN and HN conditions and found that both NUE and NUpE decreased in *wfzp* and *Tasyd* mutants, while no significant difference was observed in NUtE, compared with the WT (Figure 5I; Figure S18, Supporting Information). Taken together, these results demonstrate that the WFZP‐TaSYD module is a major determinant of NUE, primarily by affecting NUpE.

### 2.6 WFZP‐TaSYD Module Regulates Downstream Gene Expression by Modulating Chromatin Accessibility and Histone Modifications

**Figure 6:**
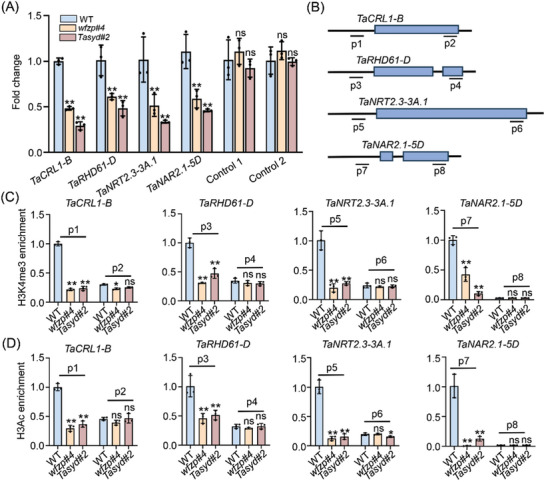
The WFZP‐TaSYD module affects chromatin accessibility and histone modification of downstream genes. A) Results of ATAC–qPCR experiments validating the chromatin accessibility changes in the downstream genes of *wfzp#4* and *Tasyd#2* mutants compared to WT. The expression levels of WT were set as 1. Controls (1 and 2) are the regions that did not change in accessible level. Values are the means ± s.d. (Student's *t*‐test, ^**^ *p* < 0.01, *n* = 3). ns, not significant. B) Diagrams of selected genes structures: Blue boxes indicate exons and long black lines represent the promoter, introns, and 3′ terminal sequences, respectively. The black lines below the gene structures represent PCR fragments tested in chromatin immunoprecipitation (ChIP)‐quantitative (q) PCR in (C, D). C,D) ChIP‐qPCR examined the H3K4me3 (C) and H3 acetylation modification level (D) of the selected genes in WT, *wfzp#4* and *Tasyd#2* mutant background. The expression levels of WT were set as 1. Values are the means ± s.d. (Student's *t*‐test, ^*^ *p* < 0.05; ^**^ *p* < 0.01, *n* = 3). ns, not significant.

Extensive studies revealed that chromatin remodelers could regulate chromatin accessibility and open chromatin‐related histone modifications,^[^ ^36^, ^37^ ^]^ thus the interaction between WFZP and TaSYD prompted us to examine the potential roles of WFZP on the nucleosome arrangement around the transcription start sites (TSS). By performing an assay for transposase‐accessible chromatin with qPCR (ATAC‐qPCR), we found that the nucleosome occupancies of downstream genes around the transcription start site (TSS) were decreased in *wfzp* and *Tasyd* mutants (**Figure** **6**A). To determine whether the WFZP‐TaSYD complex affects open chromatin‐related histone modifications, we examined H3K4me3 and H3 acetylation levels at these loci in different regions. The ChIP‐qPCR results demonstrate a significant decrease in H3K4me3 and H3 acetylation levels at these loci in the *wfzp* and *Tasyd* mutants compared to those in WT (Figure 6B–D). Taken together, the WFZP‐TaSYD module regulates root development and nitrogen uptake efficiency by increasing DNA accessibility and the deposition of H3K4me3 and H3 acetylation of downstream genes.

### 2.7 *WFZP‐A‐I* Holds the Potential for Improving LRN and TGW in Wheat

**Figure 7:**
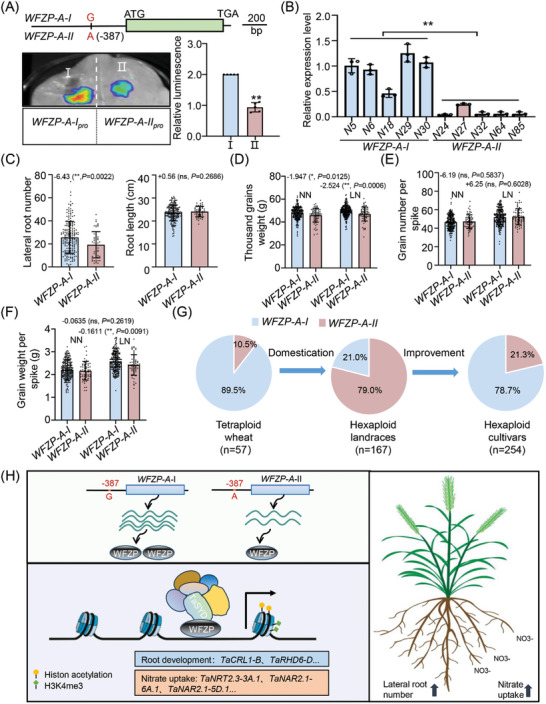
*WFZP‐A‐I* haplotype significantly increases the LRN and TGW in modern cultivars. A) The *WFZP‐A‐I* haplotype showed higher promoter activities than *WFZP‐A‐II* haplotype in *N. benthamiana* leaves. Mean values in combination I was set to 1. Values are means ± s.d. (Student's *t*‐test, ^**^ *p* < 0.01, *n* = 3). B) qRT‐PCR analyze the relative expression level of *WFZP‐A* in young root of two different haplotypes in selected accessions. Accessions’ names could be seen in supplemental Dataset S7. Values are means ± s.d. (Student's *t*‐test, ^**^ *p* < 0.01, *n* = 3). C) Statistical analysis of the LRN (left) and RL (right) between *WFZP‐I* and *WFZP‐II* haplotypes in 254 cultivars after 10 days of hydroponic growth. Values are means ± s.d. (Student's *t*‐test, ^**^ *p* < 0.01). D–F) Measurement of Thousand grain weight (D), Grain number per spike (E) and Grain weight per spike (F) of 254 cultivar accessions were analyzed by *WZP‐A‐I* haplotype under normal nitrogen (NN) and low nitrogen (LN) conditions. Values are means ± s.d. (Student's *t*‐test, ^*^ *p* <0.05; ^**^ *p* < 0.01.). ns, not significant. G) Haplotype analysis revealed that *WFZP‐A* was under human selection. n corresponds to the total number of varieties in each pool. H) A proposed working model. a variation in the 387 loci upstream of *WFZP‐A* effects the expression of *WFZP‐A*. The interaction between the WFZP protein and TaSYD directly activates genes related to root development and nitrate uptake, thereby altering the chromatin accessibility, H3K4me3 and H3AC levels of downstream genes, ultimately promoting lateral root development and nitrate uptake.

Previous studies indicated that a natural mutation in the promoter region (−387 bp) of the *WFZP‐A* is associated with the thousand‐grain weight (TGW) and the spikelet number per spike (SNS), and *WFZP‐A‐I* haplotype was a favorable allele for TGW due to the high expression of *WFZP‐A*.^[^ ^38^ ^]^ So, we wonder if this G/A allelic variation is associated with root development. First, we analyzed the promoter activities of the *WFZP‐A‐I* and *WFZP‐A‐II* haplotypes in *N. benthamiana* leaves. The results indicated that the *WFZP‐A‐I* haplotype exhibited significantly stronger expression activity than the *WFZP‐A‐II* haplotype (**Figure** **7**A). We randomly selected five accessions carrying *WFZP‐I* and five accessions carrying *WFZP‐II* to analyze the transcription levels of *WFZP‐A* in roots. The results showed that cultivars harboring *WFZP‐A‐I* haplotype had higher expression levels of *WFZP‐A* compared to cultivars with *WFZP‐A‐II* haplotype (Figure 7B). To compare the contribution of *WFZP‐A‐I* and *WFZP‐A‐II* to root development, we examined the RL and LRN of 254 hexaploid cultivars after 10 days of hydroponics. The cultivars with the *WFZP‐A‐I* haplotype exhibited a higher LRN compared to those with the *WFZP‐A‐II* haplotype, while the RL showed no significant difference (Figure 7C). This further demonstrated that *WFZP‐A‐I* is a favorite haplotype for enhancing LRN. We also conducted field trials with these cultivars under LN and NN conditions. Our results indicated that accessions harboring the *WFZP‐A‐I* haplotype exhibited a higher TGW compared to those with the *WFZP‐A‐II* haplotype under NN conditions, with no effect on the number of grains per spike or grain weight per spike. In contrast, under LN conditions, accessions with the *WFZP‐A‐I* haplotype showed both a higher TGW and grain weight per spike than those with the *WFZP‐A‐II* haplotype (Figure 7D–F). Taken together, these results indicated that fine‐tuning *WFZP* expression was a feasible and achievable strategy for further increasing LRN and TGW in cultivated wheat.

We next analyzed the frequency and distribution of the two alleles in different ploidy wheat accessions from different regions, including 56 tetraploid wheat accessions, 165 hexaploid landraces, and 254 hexaploid cultivars. In tetraploid wheat, over 89% of varieties carried the *WFZP‐A‐I* haplotype. However, during evolution and domestication, the frequency of the *WFZP‐A‐I* haplotype decreased to 21% in hexaploid landraces. Through artificial selection, the frequency of the *WFZP‐A‐I* haplotype increased to 78.7% and became predominant in hexaploid cultivars, implying that the *WFZP‐A‐I* haplotype may be beneficial for crop production and therefore be reselected (Figure 7G).

## 3 Discussion

WFZP belongs to the AP2/ERF family, which is essential for plant developmental regulators.^[^ ^39^, ^40^ ^]^ These transcription factors contained the AP2 DNA‐binding domain that directly interacts with cis‐elements, such as dehydration‐responsive elements (DRE)/C‐repeat element (CRT) and/or GCC box.^[^ ^41^, ^42^ ^]^ The previous work indicated that WFZP not only regulates the spike development, but also influences the awn elongation.^[^ ^25^ ^]^ In this work, we presented evidence that WFZP is required for the development of RL and LRN in wheat (Figures 1A–C). We found that *WFZP* is expressed in root tips and lateral root primordium (Figure 1D,E). The root growth defects caused by *WFZP* mutation and transcriptome analysis suggest that WFZP may establish a transcriptional program involved in auxin signaling to stimulate cell division in lateral roots.

Our results also suggested that WFZP directly activates the sentinel genes of root development, including *TaRHD6* and *TaCRL1*. *RHD6* in Arabidopsis encodes a basic helix loop helix transcription factor that promotes root hair development by directly activating the hair length‐determining gene *RHD6‐LIKE 4* (*RSL4*).^[^ ^43^ ^]^ *TaCRL1* encodes the orthologue of rice *Crown rootless1*, a target of an ARF factor in auxin signaling that is essential for crown root and lateral root formation.^[^ ^6^ ^]^ In addition, WFZP regulates many auxin synthesis and transport‐related genes, including *TaYUC8‐5B*, *TaPIN5b‐5D*, and *TaAUX5‐4B*, implying auxin plays an important role in WFZP‐mediated lateral root development. Root length also plays a critical role in the absorption of water and nutrients from the soil. In our study, we found that WFZP and TaSYD have a minor effect on root length development (Figure 1B; Figure 2H), suggesting that the WFZP‐TaSYD module influences various aspects of root architecture.

Nitrogen is an essential nutrient for plant growth, largely determining crop yields. In our study, we observed significant differences in nitrogen uptake rates and total nitrogen content among WT, *wfzp*, and *Tasyd*, as illustrated in Figure 5E,F. LRN and RL play a crucial role in nutrient acquisition by expanding the root surface area and enhancing soil exploration. The *wfzp* and *Tasyd* mutants exhibit reduced lateral root number and root length, which may limit their ability to effectively absorb nitrogen, resulting in lower nitrogen absorption rates compared to WT. Transcriptomic and field experiments indicate that the WFZP‐TaSYD modules can affect nitrogen uptake rates by regulating the expression of genes such as *TaNRTs* and *TaNARs*. These results suggest that WFZP has dual roles to promote nitrogen utilization, through modulating root systems and the expressions of N‐related genes. To further explore the regulation of WFZP‐TaSYD to yield and NUE in varying soil nitrogen levels, we compared the plot yield and NUE of the *wfzp* and *Tasyd* mutants with WT under both NN and LN conditions. In the *wfzp* mutants, the actual yield per plot and NUE were reduced by ≈28.4–37.8% under NN and 31.8–48.1% under LN conditions (Figure 5G,H). Similarly, the *Tasyd* mutants showed reductions of ≈8.9–9.1% under NN and ≈22.3–22.7% under LN conditions (Figure 5G,H). These findings further suggest that the regulation of yield and NUE by the WFZP‐TaSYD module is strongly influenced by soil nitrogen levels.

The SWI/SNF complexes are reported to play a critical role in regulating diverse biological functions in plants by changing the chromatin accessibility of specific regions.^[^ ^44^, ^45^ ^]^ Chromatin structure and histone modifications play a crucial role in regulating transcriptional dynamics during development and in response to environmental changes.^[^ ^46^, ^47^, ^48^ ^]^ Our results demonstrated that WFZP interacted with TaSYD and their mutants showed similar phenotypes, such as root development defects and low nitrate uptake rate. Transcriptome data also showed that WFZP and TaSYD may co‐regulate a subset of gene expression, including root development and nitrate uptake‐related genes. Based on the ATAC‐qPCR and ChIP‐qPCR assays, it was observed that the WFZP‐TaSYD module regulates downstream genes by modulating the chromatin accessibility and the levels of H3K4me3 and H3 acetylation, which are associated with active transcription (Figure 6A–D). In this process, WFZP might function as a pioneer transcription factor. However, further studies are needed to identify the specific enzymes that directly mediate H3K4me3 and H3ac modifications.

Based on these observations, the present study demonstrated that the WFZP‐TaSYD module simultaneously regulates root development and nitrate uptake efficiency in wheat (Figure 7H). This process involves enhancing DNA accessibility and the deposition of H3K4me3 and H3 acetylation levels to the genes related to these processes (Figure 7H). Therefore, our work not only broadens the comprehension of LRN and NUE regulation in crops, but also provides valuable targets for crop breeding.

## 4 Experimental Section

### Plant Materials and Growth Condition

The hexaploid wheat variety Fielder was used as a wild type. The *WFZP* knockout lines (*wfzp#2* and *wfzp#4*) and *TaSYD* knockout lines (*Tasyd #1* and *Tasyd #2*) generated by CRISPR‐Cas9 technology in Fielder background were used for further analysis. Seeds were soaked in water for 2 days at room temperature and then germinated for 1 day at 37 °C. The seedlings were transferred onto a nylon net floated in a culture box with distilled water in a glasshouse (75% humidity; 16‐h light at 22 °C and 8‐h dark at 18 °C). The natural population used to test agronomic traits was planted in the field of Handan in 2022–2023. Each of the materials was planted in 3 blocks. The wheat materials for in situ hybridization and RNA‐seq analysis were grown for 10 days in hydroponic conditions. For the field experiments, the accessions were grown in a completely randomized block design with six replicates. The field experiments were carried out as a randomized block design with two nitrogen levels (225 kg ha^−1^ net nitrogen and 0 kg ha^−1^ net nitrogen) in two blocks.

### Phenotypic Analysis

The primary RL is formed at the basal pole of the embryo after seed germination. The LRN on the primary root of wheat accessions was measured using a root scanner (Perfection 4990 Photo; EPSON, https://epson.com). The data obtained were then analyzed using Adobe Photoshop software (Adobe, https://www.adobe.com). Each genotype was subjected to three biological replicates, with a minimum of 6 seedlings per replicate. For the agronomic trait comparison, 10 individuals of every material were investigated in each block.

### Determination of ^15^N Accumulation

Wheat seedlings were first cultured in water for 7 days and then moved to an adjusted nutrient solution without NH_4_NO_3_ but with 2.5 mm KNO_3_ as the nitrogen supply. Next, the seedlings were treated with an adjusted nutrient solution without 2.5 mm KNO_3_ for 2 days. Then, the seedlings were transferred into an adjusted nutrient solution with 2.5 mm ^15^N for 24 h. Finally, the seedlings were raised in 0.1 mm CaSO_4_ for 2 min to remove the ^15^N. After, the roots and shoots were separated immediately and dried at 65 °C for 5 days. Finally, the measurement of the ^15^N was performed using a continuous‐flow isotope mass spectrometer (Vario EL III/Isoprime, Elementar, Germany).

### Total N Content Measurement

Wheat seedlings were first cultured in water for 7 days and then moved to an adjusted nutrient solution with 2.5 mm KNO_3_ as the nitrogen supply for two months. Then, the roots and shoots were separated immediately and dried at 65 °C for 5 days. The N content was determined using the Auto Kjeldahl Nitrogen Analysis System (Elementar rapid N exceed, Elementar, Germany) using the Kjeldahl method. Each measurement was subjected to three biological replicates.

### NUpE, NutE, and NUE Calculation

After drying, the aboveground portions of the plants were ground into a uniform powder at mature stages. One gram of this homogeneous powder was weighed, and the nitrogen content of the plant was determined using the micro Kjeldahl method. NUpE was calculated by dividing the total nitrogen in the shoot by the amount of nitrogen fertilizer applied. NUtE was determined by dividing the grain yield by the total nitrogen in the shoot, with the overall NUE calculated as NUE = NUpE × NUtE.

### Dynamic NUtE Measurement

To calculate the dynamic NUtE between the WT and *wfzp* mutants, wheat seedlings were first cultured in water for 7 days and then transferred to an adjusted nutrient solution containing 2.5 mm KNO_3_. Sampling was conducted at different time intervals to measure dry weight and total nitrogen content. Therefore, NUtE is calculated by dividing the dry weight by the nitrogen content in that part, represented as NUtE = DW/NC, where DW and NC refer to the dry weight and nitrogen content of that part, respectively.

### Vector Construction and Plant Transformation

For CRISPR/Cas9 genome editing, small‐guide RNAs (sgRNAs) were designed according to the sequences of *TaSYD‐A/B/D* using the E‐CRISPR Design Website (http://www.e‐crisp.org/E‐CRISP/). Two target sites were obtained and named Target 1 (GGATGGACTCAAAAGCAGCAGGG) and Target 2 (GAGCAATCACATATACTGCAAGG), respectively. The MT1T2 vector was amplified using two pairs of primers containing the sgRNAs and then cloned into the CRISPR/Cas9 vector pBUE411. The plasmid constructs were introduced into *Agrobacterium tumefaciens* strain EHA105 and subsequently transferred into Fielder using *Agrobacterium*‐mediated transformation.^[^ ^49^ ^]^

### In Situ RNA Hybridization

Young root tips and lateral root primordia were fixed in 4% (w/v) paraformaldehyde; section pretreatment, hybridization, and immunological detection were performed as described previously.^[^ ^50^ ^]^ The RNA probes that specifically targeted *WFZP* or *TaSYD* were synthesized and labeled with digoxigenin, respectively. The RNA probe sequence is listed in Supplemental Table S1.

### RNA Extraction and qRT‐PCR Analysis

Total RNA was isolated with the TRIzol reagent (Invitrogen) and cDNAs were generated using HiScript II Q RT SuperMix (Vazyme Biotech, Nanjing, China). qRT‐PCR was performed on a real‐time PCR system (CFX thermocycler; Bio‐Rad, Hercules, CA) using AceQ qPCR SYBR Green Master Mix (Vazyme Biotech, Nanjing, China). Each experiment was performed with three biological replicates. Primers are listed in Supplemental Table S1.

### Yeast Two‐Hybrid Assays

The truncated CDS sequence of *WFZP‐A* without autoactivation activity was amplified and cloned into the pGBKT7 vector as a bait and transformed into Y2H Gold yeast cells to screen a yeast cDNA library derived from the wheat roots tissues of Fielder. Library screening and plasmid isolation/sequencing were performed according to the manufacturer's instructions (Clontech). Primers are listed in Table S1 (Supporting Information) and the results of the screening yeast are listed in Table S6 (Supporting Information).

### Split Luciferase Complementation Assays

The full‐length CDS of WFZP‐A and truncated TaSYD‐B (named TaSYD‐B‐F1, TaSYD‐B‐F2, TaSYD‐B‐F3, and TaSYD‐B‐F4) were cloned into the pCAMBIA‐1300‐nLUC binary vectors or pCAMBIA‐1300‐cLUC respectively. All constructs transformed into *Agrobacterium* strain GV3101 (pSoup‐p19) and paired for agroinfiltration of *N. benthamiana* leaves. After 36–48 h of co‐infiltration, the LUC activities were analyzed using NightSHADE LB985 (Berthold Technologies, Bad Wildbad, Germany). Three biological replications were performed with similar results. Primers are listed in Table S1 (Supporting Information).

### Co‐Immunoprecipitation (Co‐IP) Assays

The CDS of *WFZP‐A* was fused to pCAMBIA1300‐GFP vector and the CDS of *TaSYD‐B‐F1* was cloned into pCAMBIA1300‐Myc vector. The resulting constructs were separately introduced into *Agrobacterium* strain GV3101 and co‐infiltrated into *N. benthamiana* leaves. Co‐IP assays were carried out as described previously.^[^ ^51^ ^]^ Total proteins of *Agrobacterium*‐infiltrated *N. benthamiana* leaves were extracted using lysis buffer (50 mm Tris‐HCl pH 7.5, 150 mm NaCl, 5 mm EDTA pH 8.0, 0.1% NP‐40, 1% TritonX‐100, 0.6 mm PMSF, 20 um MG132 with protease inhibitor cocktail, Roche, Basel, Switzerland). Lysates were incubated at 4 °C for 30 min, followed by centrifugation at 13 000 g for 20 min. The supernatants were incubated with 30 uL anti‐GFP magnetic beads at 4 °C for 2 h. Immunoprecipitated samples were separated in sodium dodecyl sulfate‐polyacrylamide gel electrophoresis (SDS‐PAGE) gels and detected using anti‐GFP (HT801‐01; TransGen Biotech, Beijing, China) and anti‐MYC (HT101‐01; TransGen Biotech) antibodies. Primers are listed in Table S1 (Supporting Information).

### Pull‐Down Assays

The GST pull‐down assay was performed as previously described with minor modifications.^[^ ^52^ ^]^ The GST or GST‐fusion proteins were separately incubated with GST beads in 1 mL binding buffer (50‐mm Tris‐HCl, pH 7.5, 100‐mm NaCl, 0.2% [v/v] glycerol and 0.6% [v/v] Triton X‐100) at 4 °C for 2 h, and then were washed four times with the same buffer. The beads were resuspended in 1 mL‐binding buffer before adding the His‐fusion prey proteins. The mixture was incubated at 4 °C for 2 h, followed by washing four times. The immunoprecipitated proteins were detected using anti‐GST (30901ES50; YEASEN Biotech) and anti‐His (30401ES50; YEASEN Biotech) antibodies, respectively. Primers are listed in Table S1 (Supporting Information).

### Bimolecular Fluorescence Complementation Assay (BiFC)

The BiFC assay was performed by using n‐YFP and c‐YFP vectors harboring the fragments encoding the N‐ and C‐terminal halves of YFP, respectively. The CDSs of WFZP‐A and TaSYD‐B were separately cloned into the n‐YFP and c‐YFP vectors, and the combinations of plasmids were transformed into the *A. tumefaciens* strain GV3101 and co‐expressed in *N. benthamiana* leaves. The YFP signal was imaged after 48 h infiltration using a confocal microscope (LSM880; Carl Zeiss, Heidenheim, Germany). The primer sequences are listed in Table S1 (Supporting Information).

### RNA‐Seq and Data Analysis

To perform RNA‐seq experiments, WT, *wfzp*, and *Tasyd* roots were collected at 10 days, and total RNA was isolated using the TRIzol reagent (Invitrogen), according to the manufacturer's instructions. The *wfzp* and *Tasyd* mutants were cultured under the same conditions and sampled in two separate batches. To ensure the reliability of the data, a control WT sample was included in each experiment. Barcoded cDNA libraries were constructed using Poly‐A Purification TruSeq library reagents and were sequenced on NovaSeq platforms (Illumina, San Diego, CA, USA). After screening and trimming, clean reads were mapped to the wheat reference genome (IWGSC REFSEQ v.1.1) using TOPHAT2 software. Bioconductor package DESEQ2 was used for the determination of the differentially expressed genes (DEGs) with estimated log_2_ (fold changes) ≥1 and *p*‐value<0.05. MORPHEUS was performed to construct heatmaps (https://software.broadinstitute.org/morpheus), and TGT was used for Gene Ontology term enrichment.^[^ ^53^ ^]^

### EMSA Assays

GST, GST‐WFZP‐A, and GST‐TaSYD‐B‐F1 proteins were synthesized by in vitro transcription and translation (Promega). Oligonucleotide probes were synthesized and labeled with biotin at their 5′ ends. EMSA was performed by using a Light Shift Chemiluminescent EMSA Kit (Thermo Fisher Scientific, Waltham, MA, USA). GST and GST‐TaDST were added to the reaction mixture (5 ng biotin‐labeled annealed oligonucleotides, 2 µL 10 × binding buffer, 1 µL 50% v/v glycerol, 1 µL 100 mm MgCl_2_, 1 µL 1 mg mL‐1 poly(dI‐dC) and 1µL 1% v/v Nonidet P‐40). The reactions were then incubated at 25 °C for 20 min, followed by electrophoretic analysis. Biotin‐labeled probe signals were detected using the kit.

### Transcriptional Activity Assays

The transcriptional activity assays were performed in *N. benthamiana* leaves as previously described.^[^ ^54^ ^]^ To generate reporters, the promoters were combined with the luciferase (LUC) reporter gene in the plant binary vector pGWB35 through Gateway reactions (Invitrogen). The reporters were separately co‐expressed with the indicated protein in *N. benthamiana* through Agrobacterium‐mediated transient transformation. LUC activities were analyzed 48 h after infiltration using NightSHADE LB 985 (Berthold Technologies, Bad Wildbad, Germany). In each experiment, 9 independent *N. benthamiana* leaves were analyzed, and three biological replications were performed with similar results. Primers are listed in Table S1 (Supporting Information).

### ATAC‐qPCR Assays

The ATAC assay was performed as previously reported.^[^ ^55^ ^]^ Briefly, the treated samples were homogenized using a razor blade in a prechilled lysis buffer (1 mL) containing 15 mm Tris‐HCl (pH 7.5), 20 mm NaCl, 80 mm KCl, 0.5 mm spermine, 5 mm β‐mercaptoethanol, and 0.2% v/v Triton X‐100. The homogenized samples were then filtered twice through miracloth and layered onto a sucrose cushion (2 ml) consisting of 20 mm Tris‐HCl (pH 8.0), 2 mm MgCl₂, 2 mm EDTA, 15 mm β‐mercaptoethanol, 1.7 m sucrose, and 0.2% v/v Triton X‐100 in a 15‐mL conical tube. The crude nuclei were purified by centrifugation at 3000 g at 4 °C for 20 min. The pellet was resuspended in 500 µL of prechilled lysis buffer. The isolated nuclei were pelleted by centrifugation at 2000 g at 4 °C for 10 min. The supernatant was discarded, leaving ≈5 µL in the tube. The nuclei were then resuspended and washed with Tris‐Mg buffer (50 µL; 10 mm Tris‐HCl, pH 8.0, 5 mm MgCl₂), followed by centrifugation again under the same conditions, with ≈10 µL remaining in the tube afterward. For the Tn5 reaction, the following components were combined using an Illumina Nextera kit (catalog number FC‐121‐1031): 10.5 µL of the nuclei sample, 12.5 µL of 2× augmentation buffer, and 2 µL of TDE1. This reaction mixture was incubated in a thermocycler at 37 °C for 30 min. The resulting augmented DNA was purified with a QIAGEN Minelute PCR Purification kit (catalog number 28 004) and eluted in 20 µL of elution buffer. Initial amplification of 10 cycles was performed using New England BioLabs Next High‐Fidelity 2× PCR Master Mix (catalog number M0541S) in 50‐µL reactions. An 8‐µL aliquot was amplified for an additional 2 cycles and analyzed on a TBE acrylamide (5% w/v) gel stained with ethidium bromide. ATAC–qPCR was performed using 2× PowerUp SYBR Green (ThermoFisher Scientific, catalog number A25742). Primers used for this analysis are shown in Table S1 (Supporting Information).

### ChIP‐qPCR Assays

The roots of WT, *wfzp#2*, and *Tasyd#1* harvested after 10 days of hydroponic cultivation were used for the ChIP. ChIP assays were performed as described previously.^[^ ^56^ ^]^ In brief, after grinding the samples in liquid nitrogen, ≈1 g of materials were crosslinked with 1% formaldehyde for 5 min. The crosslinking reaction was stopped with the addition of 0.125 m glycine for another 5 min. The chromatin was isolated and sonicated to generate DNA fragments with a size of ≈500 bp. Immunoprecipitation was carried out with protein A/G‐agarose beads (GE Healthcare, 17 152 104 010 150) conjugated to 4 µg of anti‐Flag antibody (Sigma, F1804, 1:300 dilution) overnight at 4 °C. In parallel, chromatin immunoprecipitated without antibody was used as a control. The precipitated DNA was recovered and analyzed by qRT‐PCR using the primers listed in Supplementary. Three replicates were performed for each sample. Primers are listed in Table S1 (Supporting Information).

### Statistical Analysis

The averages and standard deviations (SD) of all results were calculated. The significance of the difference was examined by a two‐tailed Student's *t*‐test (^*^ *p* < 0.05; ^**^ *p* < 0.01). For comparisons involving more than two genotypes, the Shapiro‐Wilk test was employed to check for data normality. If the data were normally distributed, statistical analyses were conducted using one‐way ANOVA followed by Tukey's HSD test. When the normality assumption was not met, the Kruskal‐Wallis test and Dunnett's post hoc test were used to compare genotypes or treatments.

## Conflict of Interest

The authors declare no conflict of interest.

## Author Contributions

D.D., Z.L., and Z.J. equally contributed to this work. Z.N., J.X., and Q.S. conceived the study and designed the experiments. D.D., Z.L., and Z.J. carried out most of the experiments. J.Y., X.Z., H.Z., L.T, Y.L, R.L., F.H, X.L., and W.K. contributed to the molecular cloning, phenotyping work, and experimental data analysis. Q.S, Y.Y, M.X, J.L., and L.C. contributed to the discussion and revised the manuscript. Z.N., J.X., and D.D. wrote the manuscript.

## Supporting information



Supporting Information

Supporting Tables

## Data Availability

The data that support the findings of this study are available in the supplementary material of this article.
