# Supplementary material for: The Transcription Factor WFZP Interacts with the Chromatin Remodeler TaSYD to Regulate Root Architecture and Nitrogen Uptake Efficiency in Wheat
Source: Adv Sci (Weinh). 2025 Feb 24;12(15):2416433. doi: 10.1002/advs.202416433 (PMC12005776; doi:10.1002/advs.202416433)
Supplement: Supplementary file 1 — Supporting Information [file ADVS-12-2416433-s001.pdf]

ADVANCED SCIENCE
Open Access

Supporting Information

for *Adv. Sci.*, DOI 10.1002/advs.202416433

The Transcription Factor WFZP Interacts with the Chromatin Remodeler TaSYD to Regulate Root Architecture and Nitrogen Uptake Efficiency in Wheat

Dejie Du, Zhaoju Li, Zihao Jiang, Jun Yuan, Xiangyu Zhang, Huanhuan Zhao, Lulu Tian, Yunjie Liu, Renhan Li, Fei He, Xiongtao Li, Wensheng Ke, Lingling Chai, Jie Liu, Mingming Xin, Yingyin Yao, Qixin Sun, Jiewen Xing and Zhongfu Ni**

Supplementary Figure S1-21

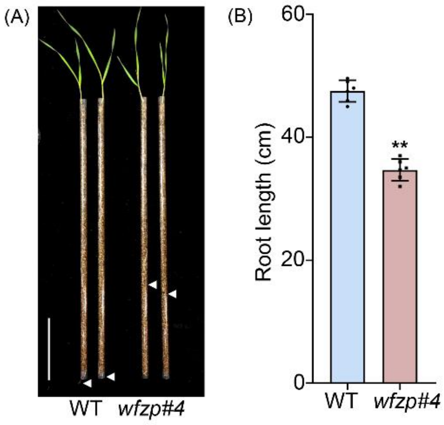

Figure S1. Root phenotypes of WT and *wfzp* mutant in seedling stage. (A) Root phenotypes of WT and *wfzp#4* grown in pipes at seeding stage. Scale bars, 10 cm. (B) Comparisons of root depth between WT and *wfzp#4*. Values are means ± s.d. (Student's *t*-test, $^{**}P < 0.01$, n = 6).

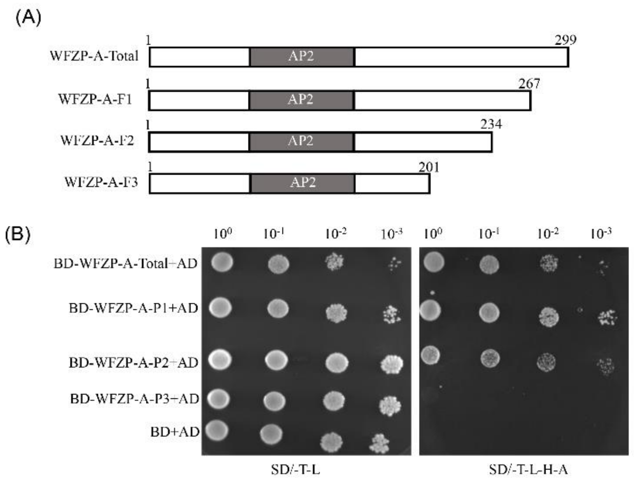

Figure S2. Transcriptional activation analysis of WFZP-A protein. (A) Schematic representation of WFZP-A and truncated WFZP-A types. The number indicated the amino acids length. (B) Transcriptional activation activity of WFZP-A and truncated WFZP-A types in yeast cells.

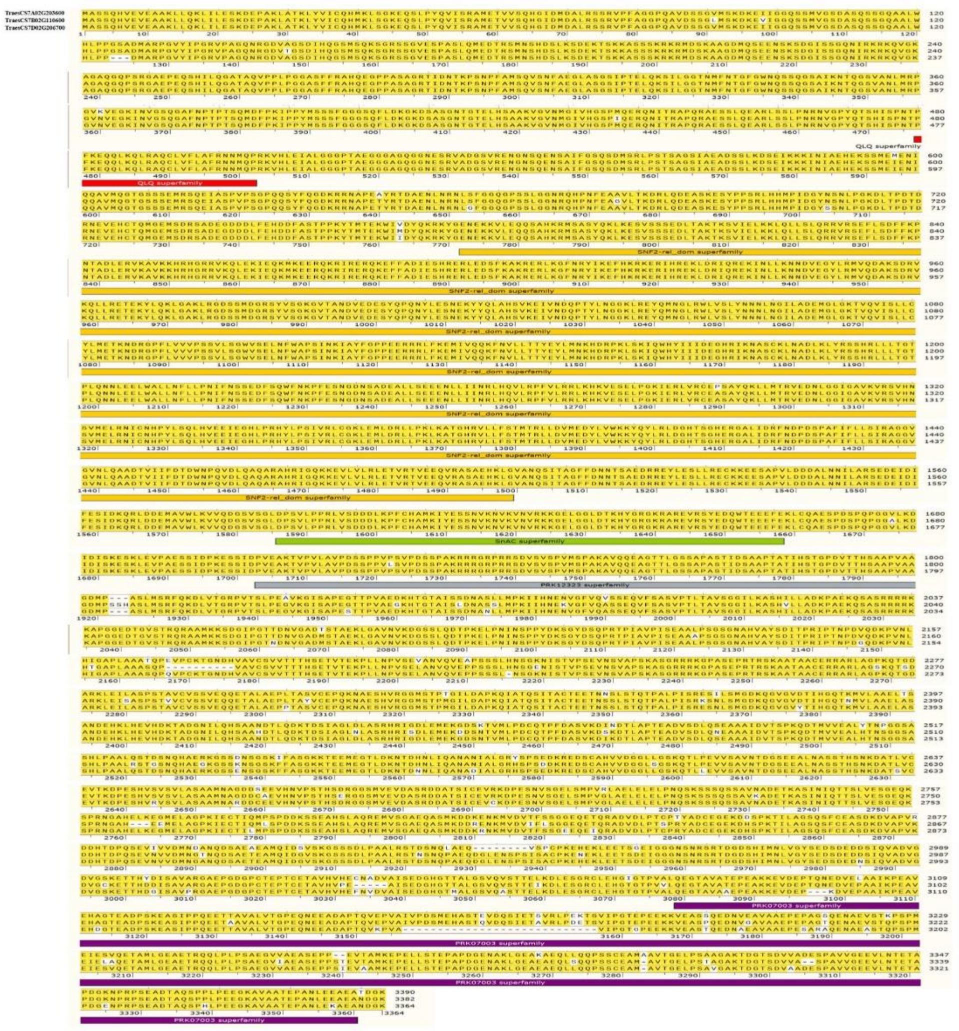

Figure S3. Protein sequences of TaSYD-A/B/D in Chinese Spring.

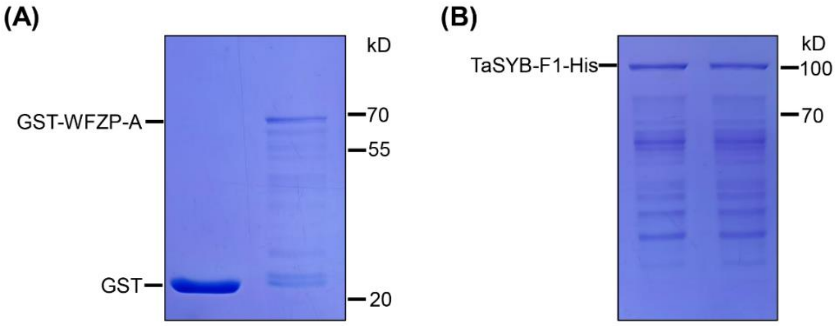

Figure S4. SDS-PAGE detection of GST-WFZP-A and TaSYB-F1-His in pull-down assay. CBB staining of GST-WFZP-A protein **(A)** and TaSYB-F1-His **(B)**. CBB, coomassie brilliant blue.

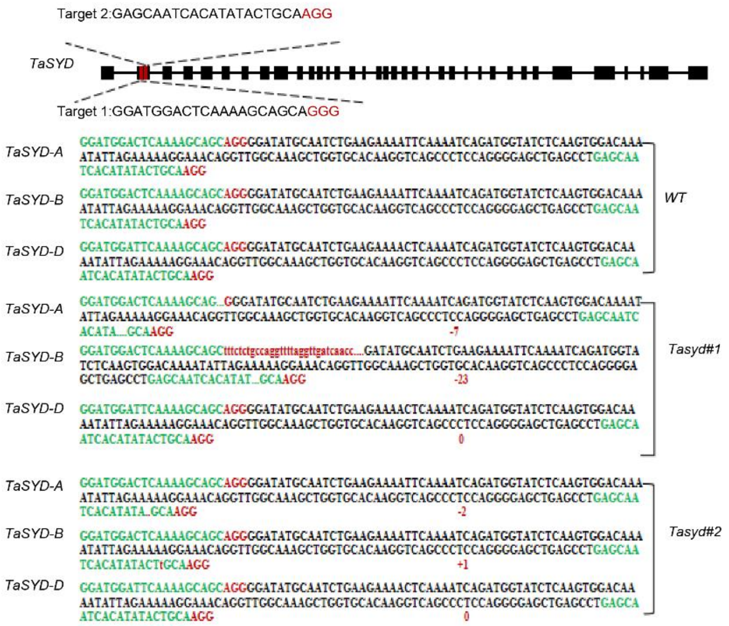

Figure S5. Generation of *Tasyd* mutants using the CRISPR/Cas9 strategy. Schematic representations of *TaSYD* gene structures from A, B and D subgenomes and the positions/sequences of target sites for gene editing. The mutation types in *TaSYD* gene loci in different *Tasyd* mutants are shown below, with target sequences and protospacer adjacent motif (PAM) sites highlighted in green and red color, respectively.

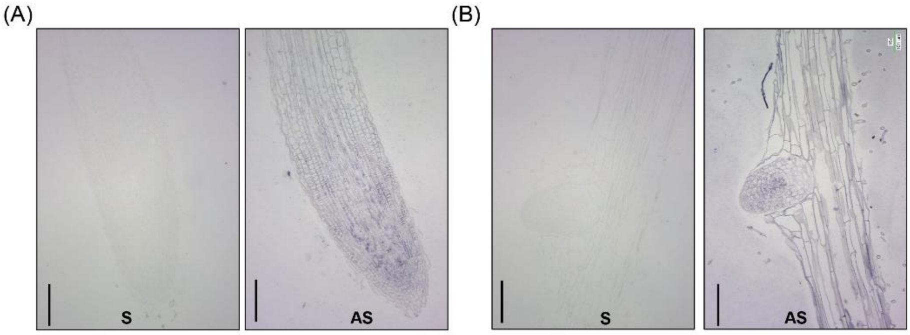

Figure S6. ***In situ*** **hybridization assays showing the transcripts of** ***TaSYD*** **in lateral root primordia.** The sense probe was used a negative control. S, the sense probe; AS, the antisense probe. Scale bars, 200μm.

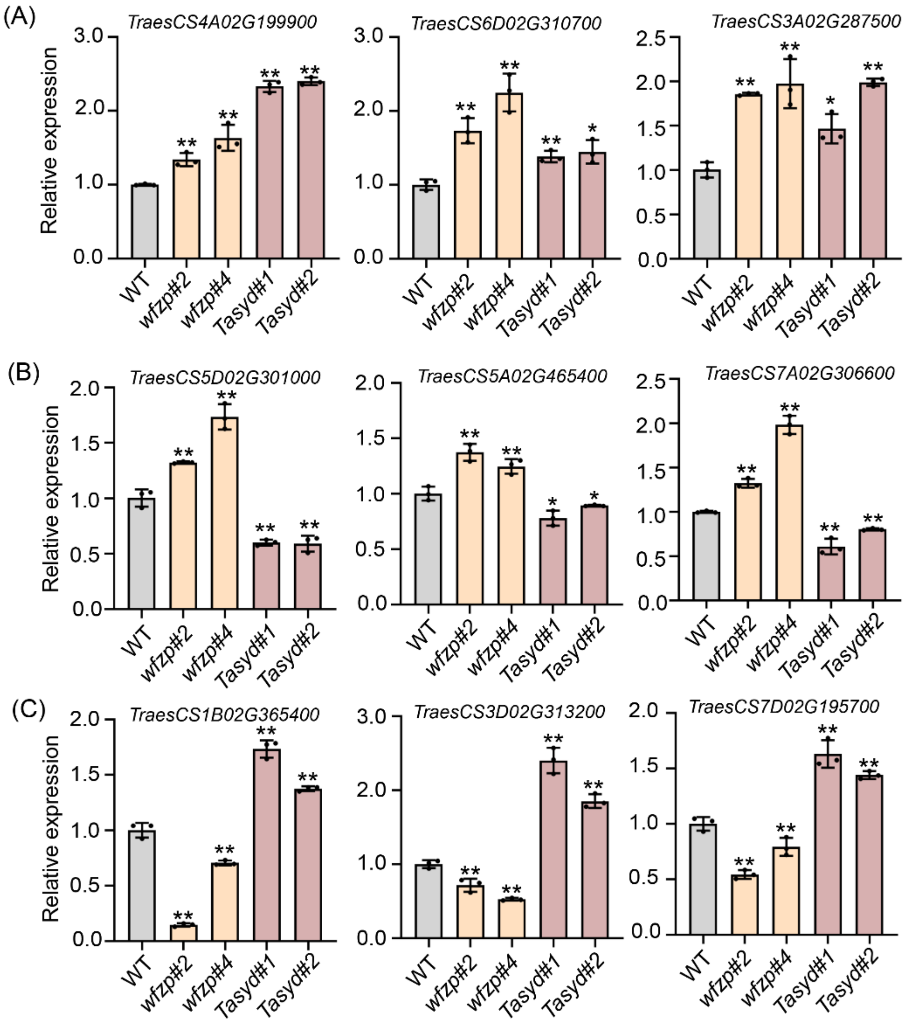

Figure S7. RT-qPCR validation of the DEGs of *wfzp* and *Tasyd* mutants.

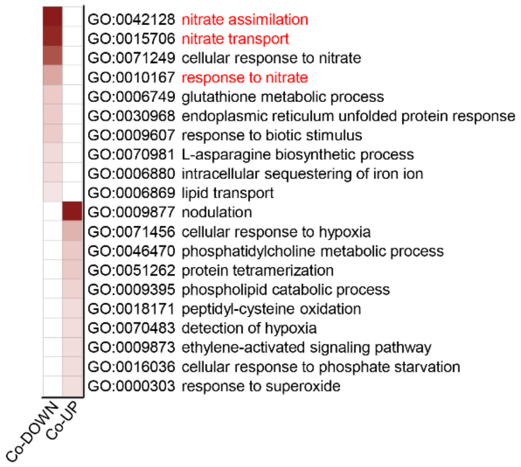

Figure S8. GO analysis of co-upregulated and co-downregulated DEGs in the *wfzp#2* and *Tasyd#1* mutants.

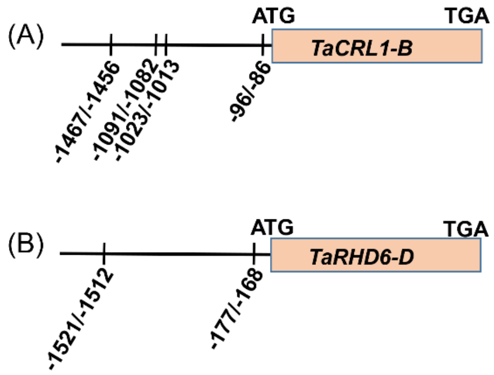

Figure S9. Schematic representations of the positions of GCC-box cis-elements in the promoters of *TaCRL1-B* and *TaRHD6-D*. The number indicated the base length.

ATCATCCGCAAATAACAGATGTGAGATACCTGGTGCTCTCCGGCAGATTTTCATGTGGTTACATCA
TTTTGCTCCTGTGTTGGTCTTGCTCCTCTTGTACCAAAATGAAAGGGCTTGTCGCCATGGCCCGA
GTGGAGCTCAAAATTAAGGTGACTCGCAGTGTCGATCTCAGAAAGATTGTAGACCTAGGGAGTAT
GACTTCGGGGTCAATGTATCCGACCTCTGCATGGCTACGGTGAGCCTGAGTCAAGACTAAAAACT
TGGTAATGAAAGGGAGTCGCGTGTTAGAATCTCATCTTTTTTGGTTTGTTGTAAGCATACAAGATAT
GTGTTGCATTAACTCTGGGTGTTTGATGTCAATGTTCCATTCATGTTGCGGTTGTCTTTTTCGTTGC
TGCAAGAGTTGACTTCTATTTTGTATGCATGCAATGATTATTTTTTCTCGACGATATGATATGTCAAT
TGCCATATGTTGCGCGAACGTGCACATGCGCGCACGCACACACACAAATGTTTCTAACGTGTGTT
CAAAATGTTGCAACAGTTTTTTTGTTTTAAATACACTGTGCAAAAAAAGGCACTACGGTGGTGATG
CAGAATGTATAAGAAATGCCGCCCTCAAAGATTTCGGGTCTGTTTGGTTGGAGACTAGTGTGGCC
AAGCCAAAGTGTGGCTAGCCACACAAGTATGGCATGCCACACAAGTGTGGCTGAGAAATTGGAC
GCCAAACTTTGGCAAAAGTTGGCAAAACAATTGTCTCTATGACAAATGGGCCATAGGGGCAATAA
AGTGTGGCAAGCCAAAGTGTGGCAAAAACCAAACACATGCCAACTAAACTGTGGCTGCCAAATT
TTGGCTTGGCAAACTGTGGCTGGGAACCAAACAGCCCCTTCATGTAGTGAGAGTCGGATGGTCC
AAATTTTGATTTCAAATAAAATTTAAGGGCGCCAAAGATAGCTAACTGTGTGTTGCCTCCATCCGG
CCGACGCCCAGCGCGCCCCTAAATGAAAAAAAAGGCATCCGTGGCTGAACATATAGTTGAGCCC
GTGGCCGACTGTGCGATGTGACGTGTGTGGGTAAGGCCCCGGGATTTTGCGCAACTGGACAAGC
ATCGGAGTAGCGGACAGAAGGCGAAGCAATAAACAAGCGGAGCAACAGACACGAAACAGAAA
AAGAAAAGGCAGAGTCCCAGCCCAGCCTCTCACCACTCCTCCTTCCCTGAAAGTGAGCGGCATC
CGGCGACAAAAGGCGTCATGCATGGCTGCTCCTTGTTTCTTTCTTCTTCTTTTTTCTTCTACGTCCC
AGCTAACACATCTCATCACACGCCACCTTCTCCGTTTCAACGTGAGCAACAGTTGTTTGAGGCCC
TCAACGTGGAATTACTACACTACAAGTCAAGCTTATGCGGCACATGTCGTCTGAAATCTCTTGACA
CTGGTACTGAGCACTGACAGTGCAGTGTGTTTCTCCCGAAAAGTATCAGCCTGAACCCTCAAATC
CAGCGGTGCTAATTTCGATGCCAGTTGTTTTTCTTCGCAAACTGGCATCAGCTCTAGTGATCGATG
ATGTTACCTAAAGATTTCTAGTAGAGAAGAACATTTCGATGGTCTAGTCCTATAATTAGCTTTCCTC
TTTCTAAGGCGATACAAGAATATATGGCGAGTTCGTCCATGATCAGTATTCATCTGATACCTGTTCT
CTCTGCTCCTTGCGAAATGTGTGAGCCGTCAATCATGCAAATCAACAGTACGCAAGGCAGACATG
TGCATTTCTCTCATTTTTCTTGGCACCAAGTTTAATCGCACTATTCAAGTAGCCGGTGTCCCCGAA
CCGCACACACGAGCCCCTCGCGCTCCTGTACATGATGCGCATTCTTCTCTGGCCCTGTCCCCTCG
CAGCCCATGCCTTCGCGTCACCTCCTCTACTCTAGTCGACCTATAAAACTCCGGTCTCGTGCGTG
TGCAGCTAACTGAAAGCGCCGTCTCCACAGGTCAGAGCTCTATCATAAGCTAGCTAGCCAGGCC
AGGCGAGCGATCGGACGATCGGGCTACAATTTCGACTACGGCGACG

Figure S10. Promoter sequences of *TaCRL1-B* in Fielder. The green shadowed area represents the location of the GCC-box.

TGATTGTCCAAGTTATAATGAAGTATTTTTTTGGCAAGTGTGCACCAATGTCCCCATGCATTGGATC
AACCACACCTCATACCCACTACCGCCCCACATAACAAGTACTCTAGAAAGACCAAATTTATTCATT
ATCTCATTTCTAGGGTGTGTAGGACAAAAAAAATTGGAAATATGAAGAGGAAGAGGAAATGAGGA
GGAAGAGCTCCATCTCACAAAATCCATGCTAGGGTGTGGTGTCTCTACTGCCTGACACCTCCTGA
GCTCCATCCCAAAATGTTGCTTCTGCGATCAAAGTGAGACTACTAAAATCTCATTTTTACTACCATT
ACTCGTCCATGGTGGCATCTAACTTATGTACTCCTTGAATCTTTCCTAACATGTTTGGTAACCGGGT
GAGATGATTAAATACAACTTCGAAGACTCTTATTGTGGCCGGATTGCGATCTCACTTTGGCTATGG
AGGAACAACATCTTTTTTCATAAGAGGAAAATTAATCTCAAATGTGAATTTTATATTCATGTAAATTT
TAGCTCCGTACTTGGTGTACAATTCAACCCATGGCGGTGGTACCCCGTCAAAGACCTGTTTACAA
GGAATGTGGGGTCAGCCTAGTCCTCACATCATGGTGTCTTGAGTGTTCTCTTCTAGACAGTTTGCT
TTCCTCTTACTTGTTGTTGGCACATTATGTTTTTGCTGGGTTTGTCTTTTTGGTTTTGTAGTACTCCC
TTCGGTCCTTTTTGCTCATGTAAGATTTGTCTGAAGTTTGACCAACTTTATAGGAAAAATATTAATAT
TCATAATATGAAACTAATATCATTAGATGTGTCATGAATTTAATTTTCATACATTATAACTTTAGAATT
GTCTACGTTGATACTTTTTATATATAAATAGGATCAAGCTTTACGTAGTTGACTTCAGACAAATCTTA
TATGCAGAGTAAAAAGGACCCGATGGAGTAAGTTGGTTGTATACATTTTAGATGATAGGCTAGGTG
AGCATCCATTTATGACAAGTTATACGGATACCAGTGTAAGAACCCATCAATAAAAGCCATCTTTTTT
TAAAGATGGATGTTATGCTACTATGAGTCATGCATGACGATAAATAAGATGATTTATGATACTAATCT
ATGATATTATGCACTATGAATGTAATATTATAGACTAGTACCATATGCATGATACTAATGTATAATACT
CCCCACTTTAAGGAACATACTAATGTTGATACTCCCTAATAACTTCTTGACACTGATGCACTGCTTC
TTGATATCTCCTGCATTTTATTTTAATTTTTTGCTTCTTGACATCGATGCTTTGATAATATGTAAATTTT
TGCATGGCCGCAAATAAAGAGAAGGCAAGGCAACTCGGCGTATGCGGCCTAGCTGACCCGCCG
CTGCATGGCATGGCCATGGAGTAATGCTACACGTACAAACAAGTTACATGGTTTTATAAAGAGGGT
TAATTTGATTGCATGCCACGTGAGAAGAGAGGGGTCGCGAATCTGCAGCGCGTGGGAGAGCCTG
TCGCCTCTGCCGCGCCTTCTGCTCCCTGCCTCCCCGCCACCCAAACGGGTCGCTGTTCCTCTTC
CTTCAGTTCTGTTGGATCCGCACTGCGCACACAATCCCTCCTTCAGTACTTCATTTCGAAGCTCCA
CTGAGTGAACACACCCGATTGCCCGATACCCGCGATGGAGTTGGCGGGCGAGTTCTTCAGGCTC
GCTCCGGGAACAGAGGAGACACAGCTACGGCAAGGATCGGCAGCTCAATCAGCTAGCTAACTC
AAGCATTTAATGCGGATGTACTTTGTATAATCCTCCCCACGCCCAGTGTTTTGTTGTACCGGCCCC
ACATGTGCCCGCCGTGGCCCGGGCCTGTGCGCCGCGGCATGTATATATAGCCGGCGCATGGCGC
GAAGGCAGCACACAGCTCGCCGCTGATCGCACAGCGCAGAGCTCGATCGAGGTGCAACACTCT
GCTAGATTACATCACATAGCTAGCTTGGTTTAGCTGCTGTCACCCACGGAAACTACTAGTACTAGC
TAGCTAGGTACTGTAGCTACGTGATCCTGATC

Figure S11. Promoter sequences of *TaRHD6-D* in Fielder. The green shadowed area represents the location of the GCC-box.

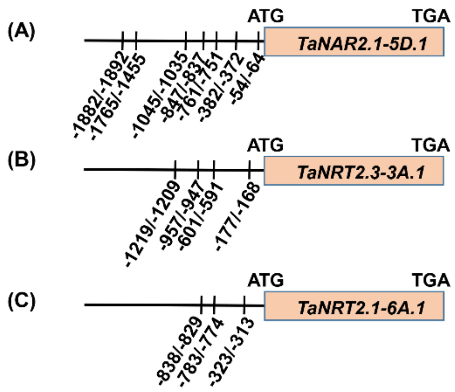

Figure S12. Schematic representations of the positions of GCC-box cis-elements in the promoters of *TaNAR2.1-5D.1*, *TaNRT2.3-3A.1* and *TaNRT2.1-6A.1*. The number indicated the base length.

CGAGCTCGCCACAACCCGTTTTCACCACTGAATGTTAACAAGACTTGTTCGAGAATGTGAGGCTA
ATCTTAAACTCTTTTTCCCCGTTGAAGAAGTACTTGAGTCCGGTTCCTCTTCAAATCACCAGGCTC
ACAATGATGTGGCTAATCGTGACCCAAAGTCATCTTTGACACAAAGAGCTAGCTCAGATGTGTAC
TGAAGCACACTCGCAGCAATGAAAGCGATAGAAGACCTTAAGAAAGCCAACTCTACTTATAATGC
CCTGTCTCTGGCTAGGTTCTTCCATGGAGAGGATGGCGAAGCTTGTTCTAGTGCTGTAACTTCTG
AGAGCTTACTGTTAGGTTCCTTGCTAGATTCCCAGGTTGAAGATGCAGATAAGGATGATTAAGAG
GTGTGAAAATGCAAAAAATGTTAAACATGTGTAAAAAGGTGTTCCTTATGTATACGAAAAATATAA
AATGTATATATAAAAAAAAGTACTCTAAACGCTCTTATATTAGTTTACAGAGGGAGTATACATCAAA
ACAATATATCTGACAAAAATGTTAATCATGTATTGAAAAAGGTTCAACATGTACAAAACAATTGTTT
AAGATGTACTTGCAAAATGTTGAACGTGTATATAAACTATTCCGATTCTATAGAAAAAAAGAAAGA
AACAGGAACACAAAAATAAACAAAGAAAAATGAAGAAATCGGAGAAAGAAACAAAGAGAATTG
AAGAAAAACGAATAGAAAATTCTTGTCCTGTGTTTAAATTATCCTCAGTTTTAATTAGTTATGCCTTA
TTTGGTTTTATAGTAGATCTTATAGGAGCTGTTTTGTTATTATAGAACCAGACCCGATAAGCTCAGG
GTCGACGGATCTGGAGGTGTAGGACTCGGATCCGCTGCTCGTCATGCCGGAGAAGGTCGAAGAT
CGCCGGACAGGAGCTTGAGTGGCGGAGGAGAGTGGAGTGAAGTGGCTAGTGTTTGATCCAGGA
GCGGATGGAGAGGAATATATGTGGGTCGGGTTGGCCAGCGGCATATCGGACCCACCCACCCGG
GCGCCCCATATCAGCCTCGGATTTGGGCTGGATACGAGGGTTGCGGTCGGCGCGGGTGTTTGAA
GCCCGTTTAAGACGCCTGTCCGGGTTAAATTTTTGTGACCGGTCGCTGACTCGGCCGTCCGCTTG
AACGTATGAGACGGGTTTGGGGCACCCGGCTGTAGCTGTAGGTGCTCTTATATTATGGGACGTAC
AAAATTTGAACCCCACATGCCAGGACGTCCTCCTTTGCCGACTCTCATTTTCCTTCTCTACGATCG
CTCAACTCGACACCTTTGTCGCTCGCATCGCTCACCCAATCCCCTTTCTTTTATCCTCCGACATTC
CATCACCGTGTGATCTTGCTCTCCAGCTTCTGCCCTCACCCTAATTGTTTCCTCGTGAGCCAAGTC
AAGCAAATAATACAGCCGTGTAACCTTTGGTTAGGGCTAAGAGTCACGTCGCCTATGCCTCGGGC
CTCTTCGTCAACGCATCCATCAACAAATTCAACATGCATATGCATATGGAAATCCGTTGAGCTTTTT
GGTTGCACTCCCTTGCAATTTTGCAACAGCATTTTCTTGTACTTGGAGGGATAAAATTTGCAACAG
CTGTTTAGGCGAACACCTTGCAACTAATTGCAGCTGTAAATAAACAAAAATAAGGCTTGACGAAT
GGTTGCGCCATGTGAACTCGGCCGAAATGCCCTCCCTCGTAGTCGGCTCGATGTCTTGTTTGGAA
CGGAAGCGCATAGAATTAGCTCAGTTAATTACTCCTGCCGCTGCTTAATTTGCAGTCGCCGCATCT
CACTCTTCCGGCCGGACCCTTTGGAGACCCTCAAGAGGATCTGACCTTCCACCACCTAATAAATT
AATTAATTAGCTTATTAATCAGATGGTTGCCTTGCCCCGGCTCCTCGACCCCAACAAGGATGGCAG
CTCCGGCTCATCGTCAACGCTCTCGATCGAGCTGCCTACATATACACGCCAAACCCATCCGTACT
CCCCCATCTCTCCCAGCCCCATCCATCAAACCGCCGCAACCTTCTCCGACCGAGCAGCAGCTGC
GAGTTCGATCGAGCCCAAGTTCGACGGCG

Figure S13. Promoter sequences *TaNAR2.1-5D.1* in Fielder. The green shadowed area represents the location of the GCC-box.

GTACCAAAGATTGCAATGCATTTTTTCTGTGGAAGCAATTGTGAAGAAGAAAAGCATAGAGCAGT
GGTGTTCTATTTTAGGGAAATTGTTGGAGACCCTCAATTTCTCATGCCCTAAAATACACTTTTCTGT
GTCCTAAATTTTATCCCGAGCAATTTTGTATGCCCTAAAATAGGGCAGCTATTAGAGATGCTTTAAG
CAACGGTCTAAAATATGTATAAGCGTCTCAAACGGGTGACGTCACACTATCATAGACAACATAGAC
ATCAAACCGTGCAATTGGATATGTACGAGTGCCGCGTTATTTTGTTTTATTGTCTCCGTCATATTTGT
CACATTGCACCTACGTACCTGGACTAAGTTCTATGTGTAAATCCAAAACCTGACGCCTCATTTGGT
TCATAGGATAGGAATTTTATAGAAATAGGAAAATCATAGGAAGTGAGGTGACATGCATTTTAATTCC
TATATAGAAGAGATGCCATTTGATGCATAGGATAGGAATTTTTCCATTGAGTCTAGGCTAATGATTTT
TTCCCTCCAAAATGTGAAGGATTGATTCCTATCCTATATAGGAATAGGAATCCATTCCTACAAACCA
AAGGGCTTCAAAGGAATTTTTCCTTTGCAAATCCTATCCTATAGAGTTCCTACAAAATTCCTACAAA
CCAAAGGAGGCCTGAATGTTTCCTAGCTGTTTGTCCGTTGGTGCAACTATAGGCGCAGTTCCCAA
CCCAGTAATGGAGGATCTCCAATCAGTCGGAGGAATATTATTTCCCTGAAACGATAGTCCTGCCG
AATACTCCTATAGGAGCGTTTTGTTTGCCTGTCTTTGGAAACGATAAATATGCCGGTGTCGTTTGG
CTGTAATCTTAGTTGTGATAGGTCCTTGATGCCGCACCATGGTTTTTTCTGTGAGCTTAGCTGTGGT
AGGTCACTGATGATCTTTTCAAATGCATTGTTGATTTGGTTAATATCTTGATTTGTTCATCCGCTGAC
GCGAGTTGTTTGTAGCAAAACGGCGGCTAGCGGCAGAACCACTGTTTGACAATCAGAAAACAAG
GGAACAAGTAGTAGTCGGAAGTTTACATTTTGGTTCCAGGCTTATATTACAGCCGTGGCGTTTCTT
TCATTCTCCAAGGTTAACAAACTCAAGGAGAATTACGGCACGATGGTGCAACTTGCAAGTCGCAT
CTAATGGAGAAAAGCCCAGATCAGAGCTTCTTTTTTTTTTTTTTGCGGGTGGAGATCAGAGCATAA
TAATCTTGAAATAGCAGAACAGGCTATGATCTTGAAAAAAACATGACAGAGGATGGCGTATGCTA
GCCAGTCCTGGTAGGAAACAAAAGCAAACTAGCAGTACATGCTTCCCGTGCGGCGGCACTCCCG
TACGGAGTTAGTAGCTGCCGCGTTGTCCGGGAGCACGCCGAAATCCCGAAAGCATTCATCGCCA
TTCGCCAATGATACTACAGTATTTCACAGATGCGGAGCAGGGGAGGGGGGAGCTGAGGCAGTTC
CACTACCCGAGGAACTCATCGACTTTGGCCTCCAAAGTCAGAGTTCACACGATCTCCTCTACCCT
TACAGCGACAGCAATACTACTACTACCAGATTACGTACGCCGTTTCAAAAAGAAAAGAAGAAAAA
CTACTAGCAGATTACCAGCTCGCGAACAAAACCCCGCGTATCTTCCTCGGAGAACCGCGACGCG
AGGGAGCGCTCCATCCACAGCGGTGCCGGCCTTCCACTGACCCCGTTCGATCACCACCGCAATC
TTTATTCATCCCCCGTTGCCCTCTCGCCGTCCCCAACCGTTGCGCTACGCCGCGCGCACCGATCG
AGCGCCGCTCCGGCTCCGGCTGCTCTATATATACTCGCCGCGCCGCTGAGACTGTACTGCATCTG
CATTACACACCAAGTTGCTAACACTACACAGGTGTAGCAGCAGCAGCAAGGTAGCTAGCTAGCT
GGTGAG

Figure S14. Promoter sequences *TaNRT2.3-3A.1* in Fielder. The green shadowed area represents the location of the GCC-box.

TCCCTGGAGAGGCTAAACCACCCTTTCAACAAGCCCTAATGCCAGTCTACTCTCCTATTTATCTAT
GGCCAGTTGGCCACACTGCGTGCAATTATGAAAACCCACTTTACAAAGACAGCGTTAAGCCAGT
GCCACCAACCTGGTTACCAATCTTGTTTAATGAAAATCGGTCAATTATTTAGCCCTCTACACTAGCT
CATCCGTCTTTTTTTGTGTATGTTTGGCTTGTACTTTTAAGCTTTGCGTAGATTAGAGAGAGATTAGT
ACTATCATAGTTGGATCACGGCGACGCAACCTGCACAAGGACAACCACCGTACTCTTTGCATACA
TGTGTAGGTGGCTAGCCATCCAATGTCTTGTGTGCAGCAACATCTTCTCAGAGTCAACTGTTTGG
ACGGTTTTGTACTTAGCAACACCAACCACTGCCTTGTGTGATGGGCCAAATATAGTAGCAAACCA
GCTGTGTAGAGAGTCAATGAGCGTCGTATAATGAAATCATGTAGTGCACTTAACAACAAACGATC
ACAATCATACAGCACATACATAATATAGCCATATAGGTTATCATATAGTAGAGTCACAGGAACGGTA
TGACAACCCTGATAAGTTCAAAGGGACAGACATGCTACGGGTCTCTCGGACCATATATATCTCATT
AGTCTGAATATATCTAAGTTAGACTTAGACCCCTGGTTTTAACCAAGGATTGAGCAGAAGCGGCA
CTGCATAAACATAACCAACCGGAAAACAAATAATGCTATACATTAATCTTGATGAGTAGATCCATC
GTGGAAATGGAAACGTTCAGGACATAAAAGCTAAACCAAAGTTTAACACTTGTAATGAAGAAGCT
ATGCCTGAAATAGCTTATGGAAGCTCTACTAACTAGTGAGATCTGAAATGACAAGAAGTTGCACC
ATACGACATAAGTCTAGACGATGACTAGCCCTGAGAACCATACCCCTCGCATTGCTCCACAGCCG
GGACTCTTCCTTTTGTTACCTGTATGTAGGTAAGGTCACCACCTGTCCCCGTCTAAGTATGTATGCT
GTCAGTGTCAAGGTTGACATGGTTGCAGCCCAATTGTGTGTGTGTGTCGCAAAGTCAGAGCATC
GACCGCGTGCAATGTACACGTCCTTTGGTTTTGAGGAAGACCGGAATGCAACATGTGTGTGTACG
TATGCGATCGCGGCCGTTGCATGGCGGCAAAAGCTGCACGCAACAAACAATCAATTTGAGCCAT
GCATGCTTCTGCTGGCCGACCTGTGGTCCGAAACAACAATCAATTTGTTTGCATTAGCAGGCAAG
TATCAAACTATCGATTCACTGCAGTCAAATTAAGCTGCAGCTTTCTCAAAATAAATAAATACTCCCT
CTGTCCCATAATATAAGAGCGCAAAAACGTTCATATATTGTGGGATGGAGAGAGTAATAAGTACTA
GGCTGCAGCTAAGCTATCAACAGAGCAGCACATGCGATTGCCAGATGCAGACAGAGCACATGCA
CGGAATCGTGCAGAGTTGCTGTTCCAGGTAACTCGAGAGGAGATCTGAAGTGAGACCGCAAGA
GAACAGAAGCTTGCAAAGTCGCAGCAAGTTTGTCCAGCTGCAAGTCGGGTGATCGCCAGCAGG
GAGGGAGGCAGATCGAGACCTGGACAGTGCATATTCTGATCTGATCCTTGGTCCATCCCACGGG
CCGAGGGCGCGGAGATGCGCGCGGCGGCGAGGTTAATCGCGCTAATTAATCCCTCTCTCGACTG
CGTTGCGCGCCCGTTGTTTAGTCCCACTTGCTGCTGCTGCCATGATTCCGTCAGATGCAATAATAG
TCTAATTCTTCTGTCTATCTGTCTTACCCTTCAGCTAACCAAGAGAGACGCAGCTTGATCCCAATG
TCAACTCCTGCACTCTACTATAAATACCGATGGATCCCGGATCCAAAGCACCAAACCACAGCCAC
CACTGCAAGTAATTAAGCTTAGCTCCAAGGAGCAAAGCAAAGAAGAAGCCTAGCTAGCTCCATC
AAGCTAGATCAGTAGATA

Figure S15. Promoter sequences *TaNRT2.1-6A.1* in Fielder. The green shadowed area represents the location of the GCC-box.

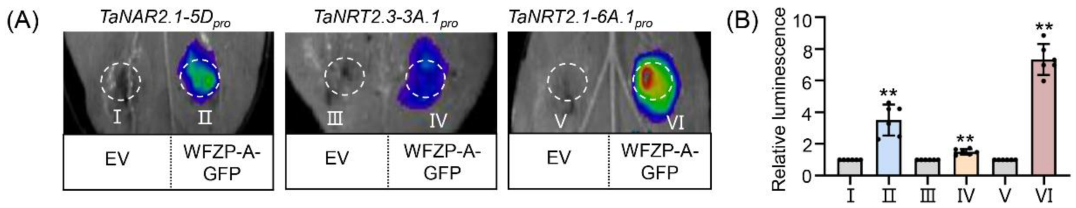

Figure S16. Transient transcriptional assays showing that WFZP significantly activates the expression of *TaNAR2.1-5D.1*, *TaNRT2.3-3A.1* and *TaNRT2.1-6A.1* genes. Left panels show representative leaf images, and the right column represents the statistical analyses of relative luminescence intensities. Values are means ± s.d. significant differences against combinations I, III and V. (Student's *t*-test, $^{**}P < 0.01$, n = 6).

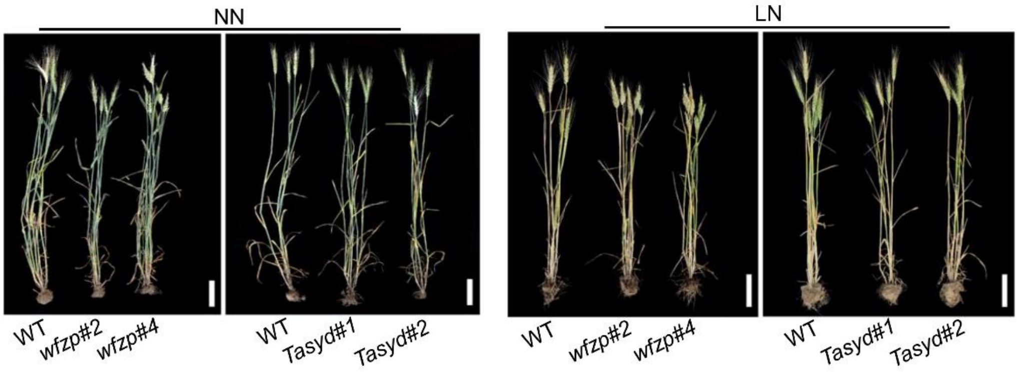

Figure S17. Growth phenotype of representative WT, *wfzp* mutants, and *Tasyd* mutants at maturing stage in the field trial. NN, normal N conditions, LN, low N conditions. Scale bars, 10 cm.

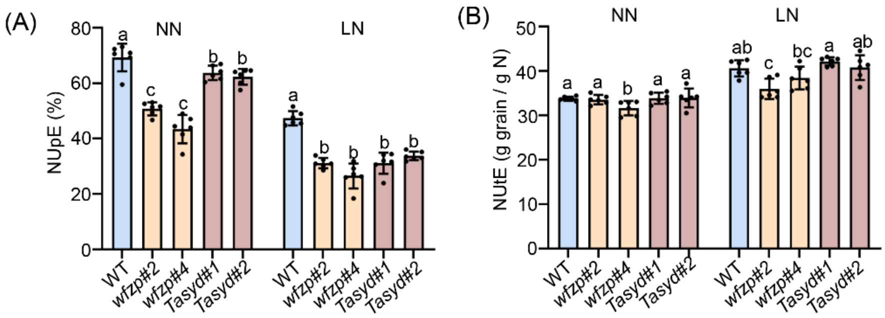

Figure S18. Nitrogen uptake efficiency (NUpE) and Nitrogen utilization efficiency (NUtE) of *wfzp* and *Tasyd* mutants under LN and HN conditions at mature stage. Nitrogen uptake efficiency (NUpE) was calculated by dividing the total nitrogen in shoot by the amount of nitrogen fertilizer. Nitrogen utilization efficiency (NUtE) was calculated by dividing the dry shoot biomass or grain yield by the total nitrogen in shoot. Values are means ±s.d. (*$P < 0.05$, n = 6, one way ANOVA, Tukey's HSD test).

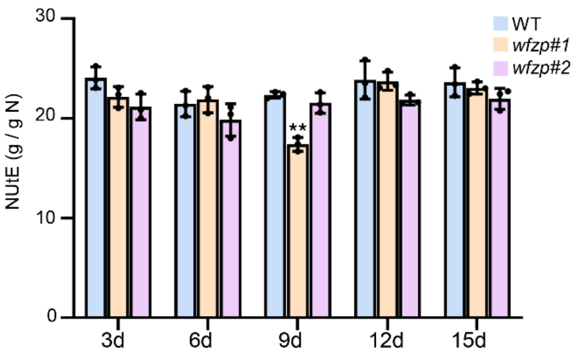

Figure S19. Comparison of dynamic nitrogen utilization efficiency (NUtE, g/g N) of *wfzp* mutants under hydroponic conditions at different time points. Values are means ± s.d. (Student's t-test, ***P* < 0.01, n = 3).

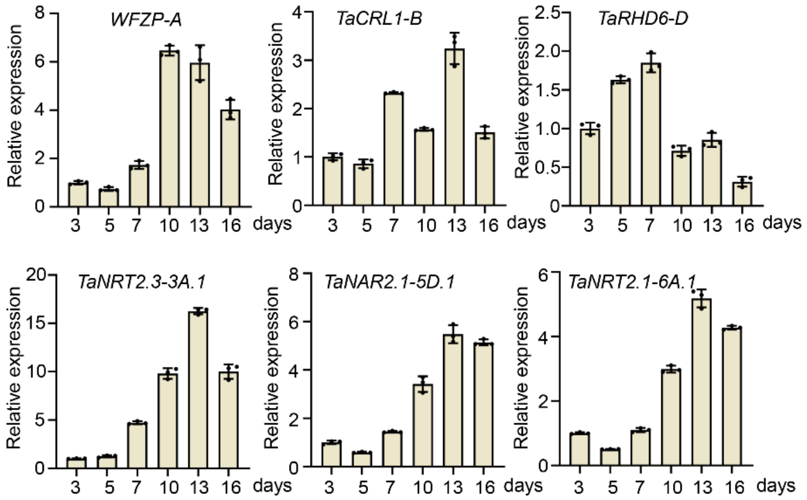

Figure S20. Expression analysis of *WFZP-A* and its downstream genes in different stages of root development.

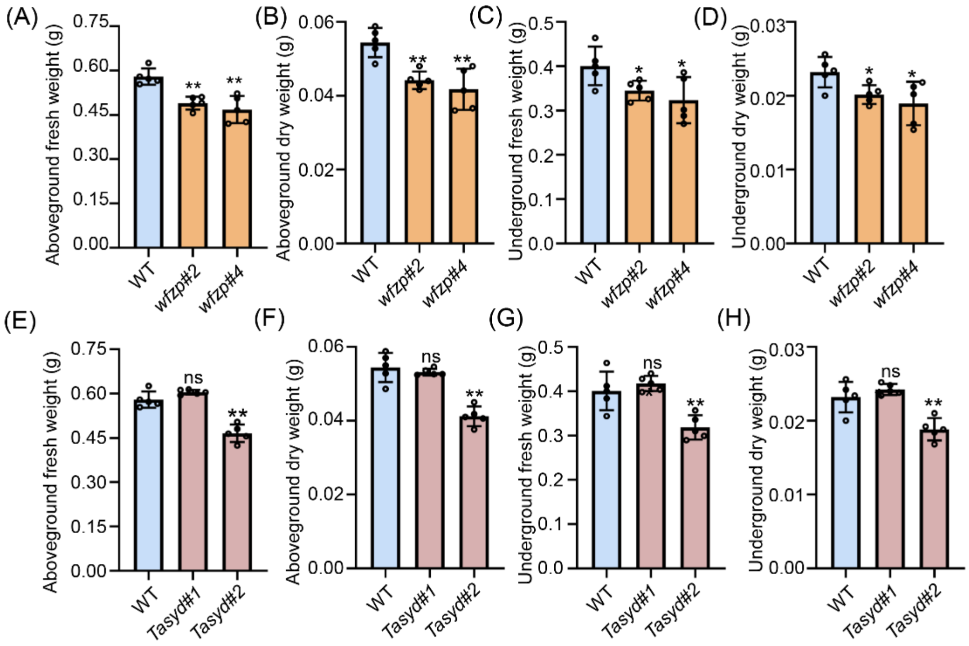

Figure S21. Phenotypic analyses of *wfzp* mutants under hydroponic conditions. (**A-D**) Measurement of the aboveground fresh weight (**A**), aboveground dry weight (**B**), underground fresh weight (**C**) and underground dry weight (**D**) of WT and *wfzp* mutants. Values are means ±s.d. (Student's *t*-test, *P<0.05, **P < 0.01, n = 5). (**E-H**) Measurement of the aboveground fresh weight (**E**), aboveground dry weight (**F**), underground fresh weight (**G**) and underground dry weight (**H**) of WT and *Tasyd* mutants. (Student's *t*-test, *P<0.05, **P < 0.01, n = 5).
